# *De Novo* DNA Synthesis in *Aedes aegypti* Midgut Cells as a Complementary Strategy to Limit Dengue Viral Replication

**DOI:** 10.3389/fmicb.2018.00801

**Published:** 2018-04-26

**Authors:** Javier Serrato-Salas, Salvador Hernández-Martínez, Jesús Martínez-Barnetche, Renaud Condé, Alejandro Alvarado-Delgado, Federico Zumaya-Estrada, Humberto Lanz-Mendoza

**Affiliations:** Centro de Investigaciones Sobre Enfermedades Infecciosas, Instituto Nacional de Salud Pública, Cuernavaca, Mexico

**Keywords:** *Aedes aegypti*, DENV, DNA endoreplication, antiviral response, *hindsight*, delta, notch

## Abstract

*Aedes aegypti* is the main vector of Dengue Virus, carrying the virus during the whole mosquito life post-infection. Few mosquito fitness costs have been associated to the virus infection, thereby allowing for a swift dissemination. In order to diminish the mosquito population, public health agency use persistent chemicals with environmental impact for disease control. Most countries barely use biological controls, if at all. With the purpose of developing novel Dengue control strategies, a detailed understanding of the unexplored virus-vector interactions is urgently needed. Damage induced (through tissue injury or bacterial invasion) DNA duplication (endoreplication) has been described in insects during epithelial cells renewal. Here, we delved into the mosquito midgut tissue ability to synthesize DNA *de novo*; postulating that Dengue virus infection could trigger a protective endoreplication mechanism in some mosquito cells. We hypothesized that the *Aedes aegypti* orthologue of the *Drosophila melanogaster hindsight* gene (not previously annotated in *Aedes aegypti* transcriptome/genome) is part of the *Delta-Notch* pathway. The activation of this transcriptional cascade leads to genomic DNA endoreplication. The amplification of the genomic copies of specific genes ultimately limits the viral spreading during infection. Conversely, inhibiting DNA synthesis capacity, hence endoreplication, leads to a higher viral replication.

## Introduction

Vector borne virus infections have recently caught the public attention with the emergence of Zika (ZIKV) and Chikungunya (CHIKV) viruses in America. Simultaneously, a re-incidence of Dengue virus (DENV) infections has been observed. Dengue infection causes a disease that present the highest mortality and morbidity rates amongst the aforementioned viruses; being the sole life-threatening virus infection. In America, over the last few years these three emergent diseases constituted an important burden to the local public health care systems. *Aedes aegypti* is the main insect vector for DENV, CHIKV, and ZV (OMS, 2009).

When the mosquitoes ingest a virus-infected blood meal, the virus reaches the midgut, invades and escapes this barrier. The tracheal and/or muscles system may also act as a viral escape conduit into the hemolymph, allowing virus spread in virtually all organs in the insect. Infection persist in most mosquito tissues during the insect lifetime. Nevertheless, the amount of viral antigen and titers can decline over time (Romoser et al., 2004; Salazar et al., 2007).

In insects, effector mechanisms like phagocytosis, nodule encapsulation, melanization, and expression of reactive oxygen species; along with antimicrobial peptides; can target and kill microorganisms. Mosquitoes ability to limit viral spreading has been found to rely on the classical insect innate immune cascades. The *Aedes aegypti* immune response acts as a key regulator for acquiring, maintaining and transmitting virus; a phenomenon known as vectorial competence. Indeed, the Toll, IMD, Jak/STAT, and RNAi signaling cascades are activated in DENV presence (Salazar et al., 2007; Xi et al., 2008; Sánchez-Vargas et al., 2009; Souza-Neto et al., 2009; Sim and Dimopoulos, 2010; Raquin and Lambrechts, 2017).

However, DENV fend off these barriers and, as a consequence, are disseminated by the mosquitoes and spread to humans (Cheng et al., 2016). This virus do repress *Aedes aegypti* antimicrobial peptide production during its infection course (Souza-Neto et al., 2009). Indeed, during bacterial challenges, DENV-infected *Aedes aegypti* cells show lowered cecropin and defensin production, when compared with virus-uninfected cells (Sim and Dimopoulos, 2010).

There exists a delicate balance between restrictive and permissive factors. Viral particles invade tissues inducing the upregulation of the host permissive factors, then the virus titer reaches a peak, and later on the host restrictive factors diminish the viral propagation. This response is tightly regulated by immune pathways and some other factors not yet explored.

In order to design alternative and complementary strategies to fight arbovirus diseases, we need to gain a better understanding of the virus-vector molecular interactions. Several transcriptomics published works showed that, during virus infection, many cell cycle genes and DNA synthesis core components are differentially expressed in mosquito tissues. The authors attribute these genes differential expression to mitochondrial stress and metabolism dysregulation (Xi et al., 2008; Behura et al., 2011; Chauhan et al., 2012; Ramirez et al., 2012; Khoo et al., 2013; Tsujimoto et al., 2017). These genes have not been attributed to a physiological response against DENV nor to endoreplication of effector genes limiting viral spreading.

Many organisms can replicate their genomes without segregating chromosomes during their immature developmental stages. This phenomenon increases the DNA content in the cells, allowing higher transcription and swifter protein output. DNA replication devoid of mitosis enhance macromolecular secretion. This process is widespread in protists, plants and many animals including arthropods, mollusks, and mammals (Edgar and Orr-Weaver, 2001; Lee et al., 2010).

In *Drosophila melanogaster*, this phenomenon has been well characterized during oocyte development. It enables the vitellogenesis and egg shell formation. Inside the egg chamber, the *Delta-Notch* interaction between follicle and nurse cells switched the cell cycle from mitosis to endocycle. The endocycle increases the number of transcription binding loci, thereby maximizing mRNA and protein synthesis in a tightly regulated process (Edgar and Orr-Weaver, 2001; Lee et al., 2010; Palmer et al., 2014).

In the fly adult midgut, the intestinal stem cells activate the *Delta-Notch* signaling in order to induce asymmetrical division to generate new stem cells and enterocytes subtypes (Guo and Ohlstein, 2015). Beside growth; stress, injury and bacteria killing are proposed to be other stimulus involved in midgut endoreplication cycles (Buchon et al., 2009; Fox and Duronio, 2013; Castagnola and Jurat-Fuentes, 2016).

In adult fly, pro-hemocytes differentiation to crystal cells is also mediated by *hindsight (hnt)* and involves *Delta-Notch* activation (Pickup et al., 2009; Terriente-Felix et al., 2013). *hnt* also functions as a regulating switch for cell differentiation in developmental and mature stages (Baechler et al., 2016).

Upon insult (puncture) *Bombyx mori* larval midgut cells endoreplicate their genomic DNA through asymmetric division without any apoptosis processes. Surrounding the wound site, the intestinal stem cells divide asymmetrically, and their enhanced DNA replication help repair damage. Cisplatin injection inhibited the post traumatic endoreplication process observed (Huang et al., 2016).

In *Anopheles albimanus*, pathogen challenges with *Plasmodium* parasite, yeast wall and dead gram-negative bacteria activate the immune system, triggering DNA endoreplication mechanism (Hernandez-Martinez et al., 2006; Hernández-Martínez et al., 2013; Contreras-Garduño et al., 2015). Insofar, the molecules involved in this process have not been thoroughly unveiled.

In this paper, we describe a DNA endoreplication *Aedes aegypti* antiviral strategy against the DENV infection.

Some DNA sequences coding for homologs of the proteins involved in *D. melanogaster* endoreplication can be found in *Aedes aegypti* transcriptome/genome. Here, we explored the presence of the main Notch canonical pathway components in *Aedes aegypti* transcriptome/genome.

We hypothesized that *Aedes aegypti* female midgut cells could enter endoreplication cycles upon insult in a similar fashion as observed in *D. melanogaster* model. We postulate that endoreplication is triggered during DENV infection, and that endoreplication limits the viral propagation through enhanced transcription of immune effectors. We tracked 5-bromo-2-deoxyuridine DNA incorporation to determine *de novo* DNA synthesis into the genome of adult mosquitoes cells. In this work, we also assess Notch transcriptional activity in mosquito midgut during DENV infection.

## Materials and methods

### Mosquitoes rearing

*Anopheles albimanus* white-striped pupal phenotype strain (Chan et al., 1994) and *Aedes aegypti* Rockefeller strain were reared in the insectary of the INSP (Instituto Nacional de Salud Pública), 100 females *Anopheles albimanus* and *Aedes aegypti* mosquitoes per group were maintained under 60–80% of relative humidity, 28 ± 1°C of environmental temperature, and 12/12 h dark/light photoperiod cycles. Adult mosquitoes were fed *ad libitum* with cotton soaked in sucrose solution (8% for *Anopheles albimanus* and 10% for *Aedes aegypti*) until blood feeding 3–5 days post-emergence. Mosquitoes were blood fed by placing an artificial glass feeder with a parafilm membrane. Mosquitoes were fed with heparinized rabbit blood for egg production. Mosquitoes were left in pans in insectary conditions until processed.

### Viral propagation and focus forming assays

DENV serotype 2 New Guinea C strain was obtained from the INSP Dengue virus collection. DENV-2 passage/amplification were realized twice in suckling mice brain and thrice in C6/36 cells. Suckling mice infected were euthanized in a CO_2_ closed-chamber according to Mexican law “Ley de Regulación Federal para el Manejo y Experimentación de Animales de la Secretaria de Agricultura, Ganadería, Desarrollo Rural, Pesca y Alimentación (SAGARPA) en la Norma Oficial NOM-062-ZOO-1999, Especificaciones técnicas para la producción, cuidado y uso de los animales de laboratorio”, and INSP internal bioethical committee approval.

C6/36 cells (ATCC-CRL 1660) were grown at 28°C in 75 cm^2^ flasks using L-15 medium (Thermo Scientific) supplemented with 10% of heat-inactivated fetal bovine serum (FBS, Byproductos). Cell monolayers were prepared seeding scrapped cells into new flasks with fresh media.

The viral stock was obtained by infecting C6/36 cells with 0.1 MOI for 7 days. DENV-2 infected and mock-infected cells were lysed by three freeze/defreeze cycles, clarified by centrifugation at 15,000 g at 4°C during 1 h, and titrated using a focus forming units assay mentioned below. Mock-infected samples consisted in C6/36 cultured in L-15 media, collected similarly (Morens et al., 1985).

LLC-MK_2_ cells (ATCC CCL-7) were propagated at 37°C in 25 cm^2^ flasks using MEM (Thermo Scientific) supplemented with 10% FBS growth media.

Viral titers were determined by Focus Forming Units Assay (FFUs). Media from the cell plates was removed and washed twice with PBS before adding 0.22 μM membrane-filtered triplicate of Log_10_ serial dilutions of the samples to be titrated. Dilutions were incubated 2 h at 37°C, and then virus-dilutions were replaced by growth media. Plates were incubated 6 days at 37°C before removing the media. Cells were fixed overnight at −20°C with methanol, then cells were rehydrated with phosphate buffer saline (PBS) pH 7.4 (137 mM NaCl, 2.7 mM KCl, 1.47 mM KH_2_PO_4_, 8.1 mM Na_2_HPO_4_, all from Sigma). Endogenous peroxidase activity was blocked by adding hydrogen peroxide 0.3% to the PBS solution. Wells were coated 1 h at 37°C with a mouse antibody against Dengue M Protein (2H2, a kindly gift of Dr. Juan Ludert, CINVESTAV, IPN) diluted 1:25 in growth media. The supernatant is then flushed and excedent media washed away. Wells are incubated 1 h at 37°C with goat anti-mouse-IgG-horseradish peroxidase (Abcam) diluted 1:1,000. DAB substrate (Thermo Scientific) is then used to reveal immunoassay. Focus were counted and titer was calculated using Reed-Muench formula described elsewhere (Baer and Kehn-Hall, 2014).

### Mosquitoes oral infection with DENV

A suspension of rabbit red blood cells was prepared as follows, 10 mL of heparinized blood were centrifuged for 5 min at 600 g, the cellular pellet was washed three times with PBS. The rabbit red blood cells were resuspended in the same volume of MEM media (Thermo Scientific). This suspension was supplemented in 50%/50% proportion with 1 × 10^8^ FFUs/mL of DENV-2 NGC. Mock control infections were prepared equally with uninfected C6/36 lysate.

Six of all the experiments were carried out using 3–5 days post-emergence *Aedes aegypti* females, with approximately 50–200 individuals per group. After oral feeding, mosquitoes were cold anesthetized (~4°C) during 5–10 min. Engorged females were placed in a new pan and maintained until processed with sucrose cotton soaked as described earlier. Biohazard residues were disposed following bioethical standard procedures.

### Mosquitoes midgut dissection, RNA and DNA extraction

Mosquitoes were anesthetized on ice and their midguts were dissected in a drop of sterile PBS. The resulting tissues were put in Trizol reagent (Thermo Scientific) for RNA extraction at −20°C. Other midgut pools were placed in Phenol-Chloroform-Isoamyl Alcohol mixture for DNA extraction. For viral titration, pools of 5 or 10 midguts were put in 50 μL of MEM and frozen at −70°C until processing.

Midgut pools placed in Trizol (Invitrogen) were homogenized using a small piston until macerated. RNA was obtained according to manufacturer's instructions and used for RT-PCR assays as per protocol.

cDNA synthesis was carried out using RevertAid Premium Transcriptase kit (Thermo Scientific) following manufacturer's instructions. Samples were saved for DNA extraction following manufacturer's instructions. To eliminate RNA traces, samples were incubated with RNAse A (Gibco) for 1 h at 37°C.

Quantitative PCR were performed using ViiA 7 Real-Time PCR System (Applied Biosystems) with the QuantStudio Real Time Software v1.3. Reactions were realized in a total volume of 10 μL containing 5 μL of SYBR Green PCR Master mix, 1.5 ng of cDNA template, 250 nmol of each one of primers, and volume completed with nuclease-free water. Reaction conditions were the next: 50°C for 2 min, 95°C for 10 min followed by 40 cycles of denaturation at 95°C for 15 s, annealing and extension at 60°C for 1 min. The Ct value obtained from the tested gene relative to the reference gene, was used to obtain delta Ct values of infected/uninfected samples, as well cisplatin/no-cisplatin treatment mentioned below. S7 gene (ribosomal unit S70) was elected as the reference gene based on previous lab published papers (Moreno-García et al., 2015; Vargas et al., 2016). Relative expression values were obtained using the delta-delta cycle threshold (DDCT) method (Bubner et al., 2004).

### DNA synthesis *de novo* in mosquitoes

Female *Anopheles albimanus* and *Aedes aegypti* mosquitoes were fed *ad libitum* with a cotton soaked in sucrose solution plus 100 μg/ml of BrdU. At day 3, mosquitoes were mock- and DENV-2 fed. Tissues samples were taken equal as kinetics infection assay mentioned above.

#### Immunofluorescence assay for BrdU incorporation

Full midgut and abdomen tissues were dissected and fixed 2 h with 4% paraformaldehyde (Sigma), fixative excess was removed from samples and permeabilized with methanol at −20°C for 10 min and washed with PBS-Tween 1% (PBS-T), samples were hydrolyzed with HCl 2N 1 h at 37°C, and neutralized by three changes of Hank's solution (NaCl 137 mM, KCl 5.4 mM, Na_2_HPO_4_ 0.25 mM, KH_2_PO_4_ 0.44 mM, CaCl2 1.3 mM, MgSO_4_ 1 mM, NaHCO_3_ 4.2 mM) for 10 min each and washed three times with PBS-T. Unspecific antibody binding was blocked 1 h at 37°C with bovine serum albumin in PBS (PBS-A). Samples were incubated overnight at 4°C with a FITC-labeled-mouse anti-BrdU monoclonal antibody (Roche). After time, samples were rinsed with PBS-T and mounted on slide using Vectashield (Vector Laboratories). Fluorescence was recorded using a photo-epifluorescente microscope (Nikon E-600), protocol was followed as it reported (Hernandez-Martinez et al., 2006; Hernández-Martínez et al., 2013; Contreras-Garduño et al., 2015).

#### ELISA assay for BrdU incorporation

For ELISA assay DNA was extracted as mentioned above, midgut gDNA samples were re-suspended in 50 μL of PBS. DNA concentration was determined measuring 230/260 nm absorbance using Nanodrop spectrophotometer. One to ten micrograms of DNA samples diluted in bicarbonate buffer pH 9.2 were transferred to a pre-treated poly-L-lysine 96 wells ELISA plate and incubated at 4°C overnight. The BrdU incorporated to the mosquito midguts cell's nucleus was determined by ELISA assay; using peroxidase conjugated monoclonal anti-BrdU antibody (substrate TMB). The optical density was recorded as 450/620 nm absorption ratio in an ELISA plate reader (Bio-Rad). DNA from sucrose fed mosquito's midguts cultivated with BrdU was used to blank readings. DNA from baker yeast grown in PBS solution mixed with 0.1 g LB powder media and BrdU 100 μg/ml (for 48 h) was used as a positive control. DNA from yeast grown without BrdU and mosquito's midguts BrdU-sucrose fed were used to normalization by assigning a value of 1 to the mean ratio.

Cisplatin (kind gift from Dr. Vicente Madrid, CISEI-INSP), a known anti-tumor agent, inhibits DNA duplication in silkworm larval midguts, without apoptotic effects (Hasinoff et al., 2004, 2005; Bragado et al., 2007; Huang et al., 2016). In order to inhibit DNA synthesis, 1 and 9-day post-infection, the *Aedes aegypti* females were fed overnight with cotton soaked with 100 μM cisplatin/sucrose solution. Effect of the cisplatin treatment was assessed through three scrutiny points: visual confirmation of ovary shape and egg bunch formation, mortality ratio and counting the number of eggs laid.

### Bio-informatics strategy

*Drosophila melanogaster* endoreplication mechanism involves the *Delta-Notch* signaling pathway, the main molecules involved in this process were obtained from KEGG^1^ and Flybase^2^ databases (Supplementary Table 1).

We searched the transcript coding for *Aedes aegypti hindsight* molecule in VectorBase database^3^, *Aedes aegypti* Liverpool Strain, AaegL3 and AaegL3.4, genome and transcriptome respectively (Giraldo-Calderón et al., 2015). FBpp0070648 peptide and FBgn0003053 nucleotide sequences from *Drosophila melanogaster* database^2^ (Gramates et al., 2017) were used as bait, screening the afore mentioned databases using BLAST program (default parameters).

To localize orthologues, OrthoDB online program was used (Zdobnov et al., 2017), and for protein properties (family, domains and repeats, gene ontology term prediction and protein architecture) InterproScan website was used (Finn et al., 2017). *In silico* translation were performed with Exonerate generic tool. Exhaustive dynamic programming algorithm was employed to find the most likely *hnt* putative coding sequence in *Aedes aegypti* genome (Slater et al., 2005). Reverse translation allowed us to find the genome coordinates of *hnt*. The complete genomic region was screened for *coding-sequences* (CDS) using mRNA prediction tools like Augustus, FGenesh, GeneID, GeneMark, and GenScan (Burge and Karlin, 1997; Salamov and Solovyev, 2000; Besemer and Borodovsky, 2005; Blanco et al., 2007; Keller et al., 2011).

### Phylogenic tree

The sequences of *hnt* and mammal orthologue *ras responding binding element-1* (Ming et al., 2013) were aligned using Multiple Sequences Alignments MUSCLE tool (Edgar, 2004).

The sequences were obtained from Vectorbase database^3^ (Anopheles albimanus AALB003334, Anopheles gambiae AGAP000984, Aedes albopictus AALF021323, Culex quinquefasciatus CPIJ017756, Stomoxys calcitrans SCAU012431, Musca domestica MDOA011777, Glossina morsitans GMOY008594, Glossina brevipalpis GBRI040699); Flybase database^2^ (Drosophila melanogaster FBgn0003053), Ensembl database^4^ (Apis mellifera GB51515, Tribolium castaneum TC009560), NCBI database^5^ (Bombus terrestris nuccore/1185570083, *Lucilia cuprina* protein/906472602, *Homo sapiens* protein/51173735, *Mus musculus* protein/85719305.

Phylogenetic tree was constructed using PhyML software v3.0 running an algorithm of maximum-likelihood allowing 1,000 bootstrap sample repetitions (Guindon et al., 2010; Lefort et al., 2017), the protein tree was visualized and optimized using FigTree tool v1.4.3^6^.

Sequences obtained and aligned were subjected to Blast2GO for Gene Ontology Terms and Interpro domains features.

### Putative protein architecture and domain features determination

Deduced *Hnt* and *RREB-1* aminoacidic sequences were analyzed with Interpro web tool (Finn et al., 2017), families and domains from the sequences were aligned to construct a visual signature of conserved features like zinc finger, zinc finger like and zinc finger-DNA binding domain, GBlocks tool was used to delete poorly aligned positions and divergent regions of the sequences (Castresana, 2000; Dereeper et al., 2008). A visual representation of the four main clusters with double zinc finger domain was made (Ming et al., 2013).

### Primer design

NCBI Primer design tool was used to search for candidate primers for amplification of part of the *Aedes aegypti hnt* putative coding regions (Ye et al., 2012), Oligo Analyzer online tool was checked for physicochemical properties (Owczarzy et al., 2008). *Hnt* PCR products were sequenced and aligned to confirm *Aedes aegypti hnt* putative coding region.

Ribosomal protein *S7* (AAEL009496) 292 bps amplicon; forward 5′ GGG ACA AAT CGG CCA GGC TAT C 3′, reverse 5′ TCG TGG ACG CTT CTG CTT GTT G 3′ primers were used for internal control PCR (Xi et al., 2008).

From CDS and mRNA predicted sequences, following primers were designed: *hnt.B* (Fwd): 5′ CGC AAG GAG TTA GAG CGT GA 3′, *hnt.B* (Rev): 5′ GTG TCG ATC GCA GTT GGA CT 3′; *hnt.C* (Fwd): 5′ AGT CCA ACT GCG ATC GAC AC 3′, *hnt.C* (Rev): 5′ CTT TCC ACC CCG ACA ACC TT 3′). *Aedes aegypti* L-4 larvae total RNA was used to perform *hnt* RT-PCR endpoint assay and PCR product nucleotide sequencing in Synthesis Unit and Sequencing of Institute of Biotechnology, UNAM. México.

The primers designed to amplify DENV universal region were DENV_all (Fwd) 5′- CAA TAT GCT GAA ACG CGA GAG AA- 3′, DENV_all (Rev) 5′- CCC CAT CTA TTC AGA ATC CCT GC−3′ for a 171 bps amplicon. A third primer was used to amplify DENV serotype 2 5′- TGC TGT TGG TGG GAT TGT TA−3′ for a 150 bps amplicon. The quantitative PCR of DENV-2 infection were normalized using a quantified plasmid with an insert of DENV-2 genome as template (kindly donated by Dr. Rosa Ma. Del Angel, CINVESTAV, IPN).

### Statistical analysis

Infection kinetics assays were performed six times. Data from all quantitative assays were subjected to D' Agostino-Pearson and Shapiro-Wilk normality tests, if positive parametric comparisons to test differences between infected/non-infected samples, One-way ANOVA and, when required, Geisser-Greenhouse's correction was used (to eliminate possible data sphericity). Data unpaired assessments Student's T with Welch's correction for infection kinetics was performed.

Non-parametric assays were Kruskal-Wallis and Mann-Whitney tests, data were verified with Kolmogorov-Smirnov test, for robust analysis. All data were subjected to a *P*-value < 0.05 for a significative difference. Data were analyzed, and graphics made in GraphPad Prism v6.01.

## Results

### Dengue virus feeding induce DNA synthesis in *Anopheles albimanus* and *Aedes aegypti*

DNA synthesis *de novo* has been observed in *Anopheles albimanus* exposed to diverse bacterial, yeast and protozoarious immune challenges (Hernandez-Martinez et al., 2006; Hernández-Martínez et al., 2013; Contreras-Garduño et al., 2015). We challenged the two mosquitoes with DENV, using the BrdU incorporation as *de novo* DNA synthesis marker. We identified midgut cells undergoing DNA synthesis phase without entering mitosis phase. The midgut and abdomen cells respond to the viral challenge initiating DNA synthesis.

**Figure 1:**
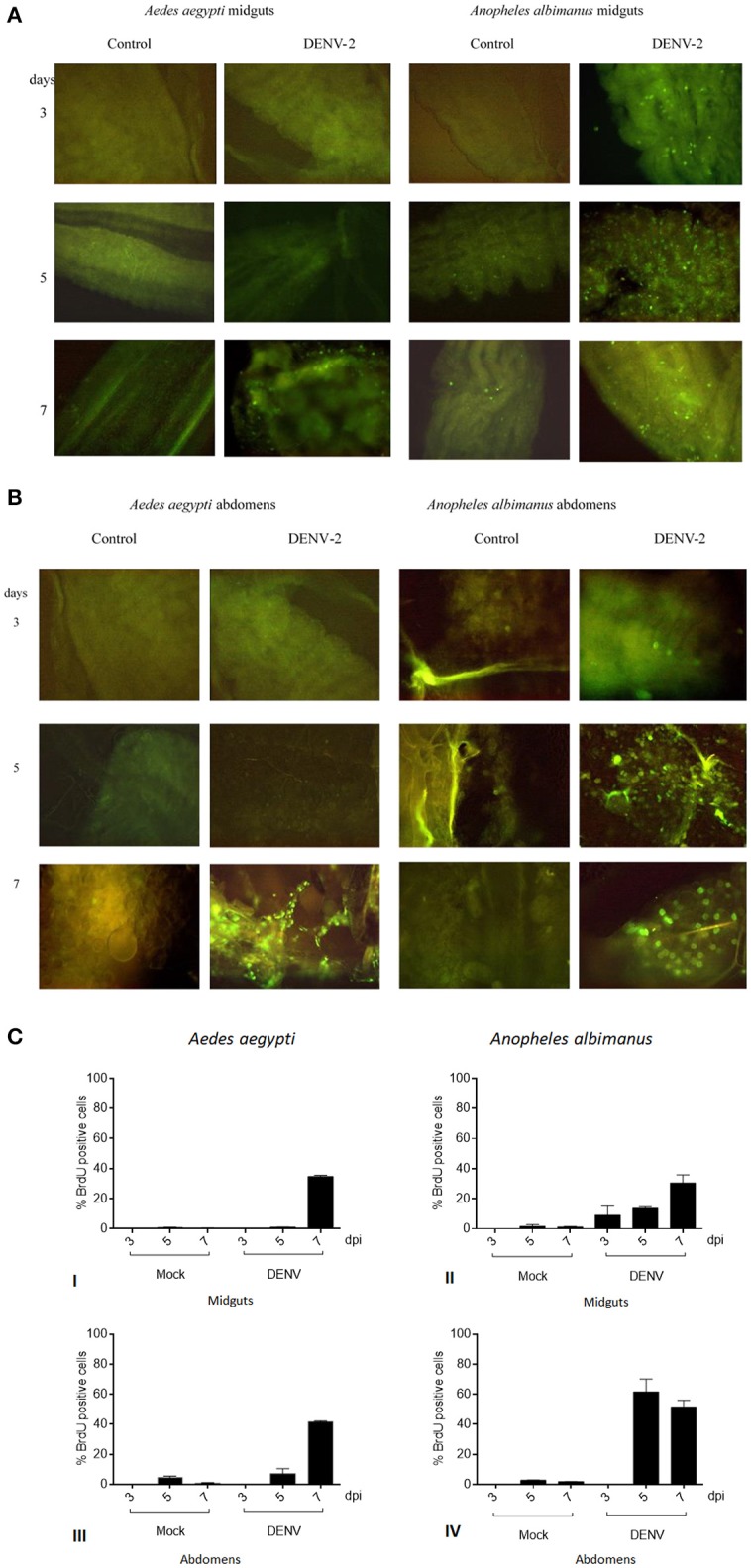
**(A)** DNA synthesis in mosquitoes challenged with Dengue virus by *in situ* immunofluorescence assay. Viral challenged tissues respond by endoreplicating genomic DNA. Control challenged tissues show no evidence of DNA synthesis. *Anopheles albimanus* was used as a positive control for DNA synthesis. Intense DNA synthesis is observed in *Anopheles albimanus* at earlier times, meanwhile *Aedes aegypti* response delays until 7 days. *Aedes aegypti* midguts control (erythrocytes plus MEM media) and DENV challenged. *Anopheles albimanus* midguts control and DENV challenged. **(B)** DNA synthesis in mosquitoes challenged with Dengue virus by *in situ* immunofluorescence assay. *Aedes aegypti* abdomens control (erythrocytes plus MEM media) and DENV challenged. *Anopheles albimanus* abdomens control and DENV challenged. **(C)** DNA synthesis in mosquitoes challenged with Dengue virus by *in situ* immunofluorescence assay. BrdU positive cell percentage per microscope field: of *Aedes aegypti* midgut cells (I) or abdomen cells (III) 3, 5, and 7 days post blood (mock) or blood plus 1 × 10^8^ FFU/mL DENV alimentation. BrdU positive cell percentage per microscope field: of *Anopheles albimanus* midgut cells (II) or abdomen cells (IV) 3, 5, and 7 days post blood (mock) or blood plus 1 × 10^8^ FFU/mL DENV alimentation.

We tracked BrdU fluorescence upon viral challenge in *Anopheles albimanus* and *Aedes aegypti* mosquitoes during seven days. Since *Anopheles albimanus* (a non-Dengue virus vector) DNA synthesis during pathogen insult has been previously described, we used this insect as a comparative model organism for BrdU incorporation. When challenged with Dengue virus, both *Anopheles albimanus* and *Aedes aegypti* mosquitoes were incorporating BrdU into their midguts and abdomens nuclei. We observed BrdU nuclear signal in *Anopheles albimanus* and *Aedes aegypti*, 5 and 7 days post virus alimentation respectively (Figures 1A,B).

*Aedes aegypti* BrdU positive midgut cells proportion in the distinct treatments were the following: at 3 and 5 days post infection, BrdU positive cells number were virtually identical in Mock and DENV fed *Aedes aegypti* intestines. Seven days post alimentation, DENV fed mosquitoes intestines showed a steep increase in BrdU intestine positive cells (35% of the cell were positive) while blood fed control mosquitoes showed no such increase (1% of labeled cells). The proportion of BrdU positive cells in the whole abdomen followed the same tendency [DENV/Mock 3 dpi (0/0), 5 dpi (7/5), 7 dpi (41/1)], showing an even larger proportion of positive cells seven days post infection (Figure 1C).

*Anopheles albimanus* is a mosquito capable to endoreplicate genomic DNA to fight fungi, bacterial and parasite infections (Hernandez-Martinez et al., 2006; Hernández-Martínez et al., 2013; Contreras-Garduño et al., 2015). DENV does not infect naturally *Anopheles albimanus* (Ramos-Castañeda et al., 2008). *Anopheline* mosquitoes natural refractoriness mechanisms to DENV are unknown. Nevertheless, it is noteworthy that DENV challenge induced a faster DNA synthesis in *Anopheles albimanus* (3 days post-challenge) than in *Aedes aegypti* (7 days post-challenge).

### *Aedes aegypti* infections with dengue virus triggers DNA synthesis

**Figure 2:**
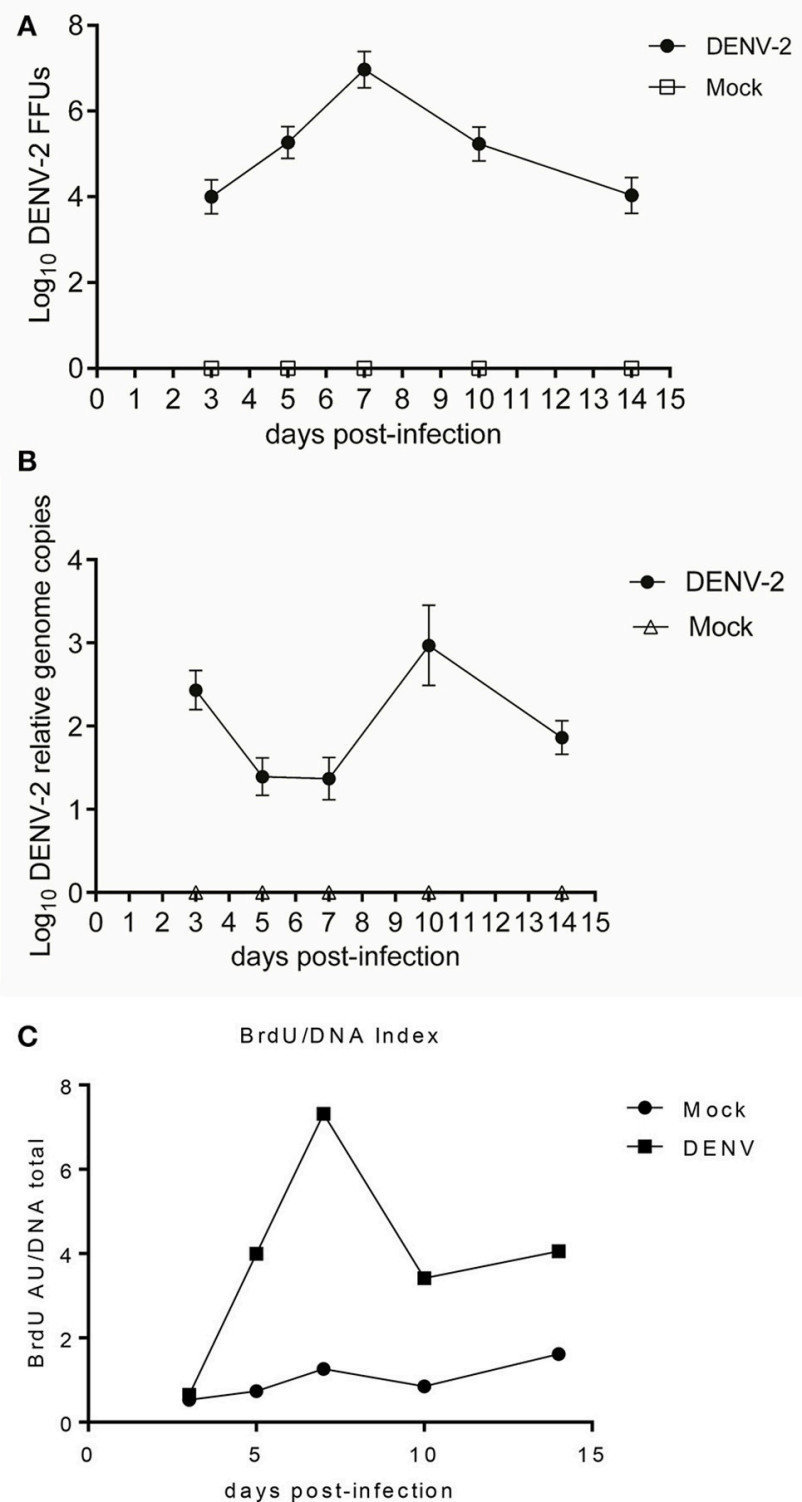
**(A)** DENV-2 and mock-infected midguts kinetics at 3, 5, 7, 10, and 14 days post-infectious blood meal. Focus forming units assay. One-way ANOVA *F* = 886.0, *p* < 0.0001. DENV-2 vs. Mock Student's *T*-test with two-tailed Welch's correction: Midguts FFU of one mosquito 3, 5, 7, 10, and 14 dpi. All comparisons had statistically significant difference *t* = 30.47, 43.0, 49.25, 39.56, 29.13; *p* < 0.0001. **(B)** DENV-2 and mock-infected midguts kinetics at 3, 5, 7, 10, and 14 days post-infectious blood meal. Viral genome copies from DENV-2 and mock-infected midguts lysate. One-way ANOVA *F* = 299.5, *p* < 0.0001. DENV-2 vs. Mock Student's *T*-test with Welch's correction two-tailed: 3, 5, 7, 10, and 14 dpi all comparisons with significant difference *t* = 30.91, 18.49, 16.27, 18.49, and 27.71; *p* < 0.0001. **(C)** DENV-2 and mock-infected midguts kinetics at 3, 5, 7, 10, and 14 days post-infectious blood meal. Kinetic of the Relative BrdU absorbtion/DNA quantity index. We determined the ratio between BrdU signal and total DNA amount, for the DENV-2 and mock-infected midguts over 14 days post infectious/mock blood meal. ELISA for BrdU incorporation. Kruskal-Wallis test = 72.83, *p* < 0.0001, DENV-2 vs. Mock Mann Whitney Test two-tailed: 3 dpi no statistically significant differences = 0.22, *p* = 0.1144; 5, 7, 10, and 14 dpi comparisons with statistically significant differences = 6.83, 10.16, 6.36, 5.19; *p* = 0.0002.

*Aedes aegypti* were fed with BrdU and 3 days later infected with DENV-2. A kinetics experiment was performed dissecting the mosquitoes 3, 5, 7, 10, and 14 days post infection/alimentation (control). The midguts of the infected mosquitoes showed a peak DNA synthesis 7 days post infection, corresponding to the point of highest viral load, as shown by FFU assay and viral genome copies (Figures 2A–C). Nevertheless, by day 10 post alimentation; 3 days after the DNA synthesis peak; the viral load diminishes.

### *In Silico* search for delta-notch signaling orthologs in *Aedes aegypti*

In *Drosophila melanogaster*, endoreplication mechanism involves the *Delta-Notch* signaling pathway. In the fruit fly, the DNA replication has been amply described during oogenesis. More recently this phenomenon has been observed in the midgut during stress challenges. The Notch signal transduction cascade is responsible for the endocycles observed in the *Drosophila* midgut cells (Lee et al., 2010; Edgar et al., 2014).

Therefore, we searched for *Aedes aegypti* orthologs of the known *D. melanogaster* proteins involved in the Notch canonical pathway. Aside from *Hindsight (hnt)* gene, all the Notch canonical pathway main proteins were identified in *Aedes aegypti* transcriptome (Supplementary Table 1).

*Hindsight* is responsible for the transition from mitotic cycle to endocycle, degrading cytoplasmic cyclins and also functioning as a transcriptional factor (Yip et al., 1997; Baechler et al., 2016). Using the peptidic sequence of *Drosophila hnt* as bait, we performed an *in-silico* translation corresponding to *Aedes aegypti hnt*. Positive matches were found in the supercontigs 1.787 and 1.430 regions, formerly described as non-coding.

We used exhaustive dynamic programming of the Exonerate generic tool to obtain an *Aedes aegypti hnt* putative protein. Reverse translation of the putative *hnt* protein allowed us to map the gene coordinates in the supercontigs 1.787 and 1.430 (Supplementary Figure 1).

**Figure 3:**
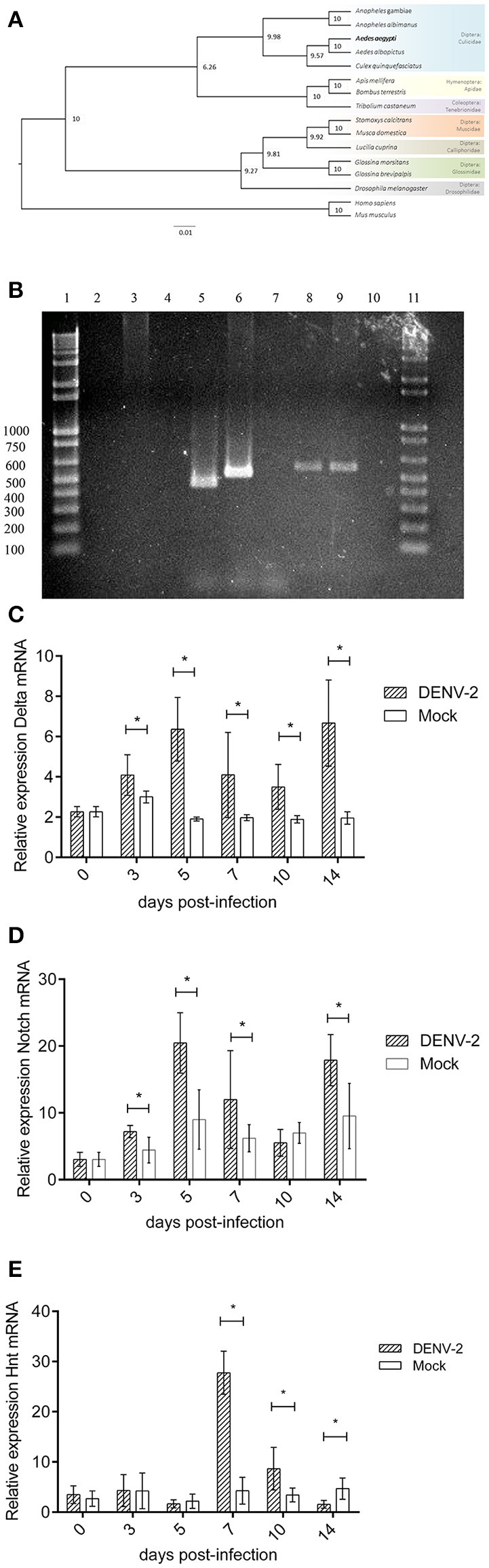
**(A)** Notch pathway transcriptional analysis during DENV infection in *Aedes aegypti* midguts. *Hindsight* and RREB-1 phylogenetic tree based on maximum-likelihood algorithm. The sequences shown were aligned using MUSCLE tool. The phylogenetic tree was constructed using PhyML software v3.0 with 1,000 bootstrap sample repetitions. **(B)** Notch pathway transcriptional analysis during DENV infection in *Aedes aegypti* midguts. Hindsight PCR from mosquito midguts gDNA and cDNA. Lanes, (1) 3 Kb Ladder. (2) cDNA (mRNA)–Hnt A amplicon. (3). gDNA–Hnt A amplicon. (4) No template control–Hnt A amplicon. (5) cDNA (mRNA)–Hnt B amplicon. (6) gDNA–Hnt B amplicon. (7) No template control–Hnt B amplicon. (8) cDNA (mRNA)–Hnt C amplicon. (9) gDNA–Hnt C amplicon. (10) No template control–Hnt C amplicon. (11) 3 Kb Ladder. **(C)** Notch pathway transcriptional analysis during DENV infection in *Aedes aegypti* midguts. Relative expression of *Delta* transcript 0, 3, 5, 7, 10, and 14 days post alimentation with blood (Mock) or blood with 1 × 10^8^ DENV2 FFU/mL (DENV-2). Mock vs. DENV-2 Student's *T*-test with Welch's correction 0, 3, 5, 7, 10, 14 dpi *t* = 0.0, 3.58, 9.74, 3.47, 4.93, and 7.55, *p* > 0.9999, 0.0055, <0.0001, 0.0082, 0.0010, <0.0001. **(D)** Notch pathway transcriptional analysis during DENV infection in *Aedes aegypti* midguts. Relative expression of *Notch* transcript 0, 3, 5, 7, 10, and 14 days post alimentation with blood (Mock) or blood with 1 × 10^8^ DENV2 FFU/mL (DENV-2). Mock vs. DENV-2 Student's *T*-test with Welch's correction 0, 3, 5, 7, 10, 14 dpi *t* = 0.0, 4.51, 6.25, 2.63, 2.04, 4.66, *p* > 0.9999, 0.0008, <0.0001, 0.0267, 0.0592, 0.0003. **(E)** Notch pathway transcriptional analysis during DENV infection in *Aedes aegypti* midguts. Relative expression of *Hindsight* transcript 0, 3, 5, 7, 10, and 14 days post alimentation with blood (Mock) or blood with 1 × 10^8^ DENV2 FFU/mL (DENV-2). Mock vs. DENV-2 Student's *T*-test with Welch's correction 0, 3, 5, 7, 10, and 14 dpi *t* = 1.18, 0.29, 1.03, 16.04, 4.06, 4.77, *p* = 0.2539, 0.7749, 0.3191, <0.0001, 0.0024, 0.0007.

The sequences of invertebrate *hnt and* vertebrate orthologue *RREB-1* were aligned using MUSCLE tool (Edgar, 2004). A phylogenetic tree was constructed (Figure 3A). The Culicid family grouped in one clade, other insects (bee, bumblebee, beetle) grouped in another clade (arthropods), and flies grouped in third clade (Guindon et al., 2010; Lefort et al., 2017). This tree shows the domains of this protein that are genuinely conserved between those species. Blast2GO output file shows that all *hnt/ RREB-1* proteins share the same putative function. Results data were consistent in GO terms (Supplementary Data Sheet 1). The *Hnt*/*RREB-1* protein family requires the presence of four zinc-finger cluster doublets in order to bind to its DNA elements (Ming et al., 2013). A map detailing the protein architecture is shown in Supplementary Figure 2.

An exon prediction analysis was made using five different tools: Augustus (Keller et al., 2011), FGenesh (Salamov and Solovyev, 2000), GeneID (Blanco et al., 2007), GeneMark (Besemer and Borodovsky, 2005) and GenScan (Burge and Karlin, 1997). *Hnt* CDS and mRNA coding regions were deduced and oligonucleotide primers for *hnt* amplification were designed, sequences in Supplementary Tables 2, 3 (Zanchi et al., 2011).

### *Aedes aegypti* Hnt, delta, and notch transcription levels during DENV infection

The *Hnt* PCR results of the mosquito genomic DNA and the cDNAs of the different experimental conditions are congruent with the *in silico* analysis results: the sizes of the respective PCR (genomic and cDNA) are consistent with the processing of the predicted intron (Figure 3B). Considering that we detected a *Hnt* transcript suitable for translation, this result further sustains the existence of the putative *Hnt* protein.

We analyzed *Hnt, Delta and Notch* transcription through quantitative RT-PCR experiments (Figures 3C–E). 3 days post-infection we observed that *Delta* and *Notch* transcriptions increase and remain higher than in mock-infected samples. This might be reflecting the onset of a strategy to prepare the DNA replication machinery for later activation. *Hnt* gene showed a peak transcription 7 days post-infection and remains over-transcribed until the 10th day. On the 14th day, *Hnt* transcription decreases to the levels of mock-infected mosquitoes (Figure 3D). The phasing of those events: first the viral challenge, then the transcription increased of those three genes, and finally the *de novo* DNA synthesis support the assumption of a causal link between those events. Also, the DNA synthesis is concomitant to virus load abatement, suggesting that either the DNA replication or the *hnt* protein synthesis are favoring antiviral activity.

### DNA synthesis inhibition affect the susceptibility of the *Aedes aegypti* mosquito to dengue

Cisplatin is a known anti-cancer agent, preferentially binding to guanine base. It interferes with DNA replication forming crosslinked DNA adducts. At high cisplatin doses (100 mM), inhibition of DNA replication leads to apoptosis of human cancerous cell line (Hasinoff et al., 2004). At low doses (100 μM), it also inhibits DNA duplication in *Bombyx mori* (Huang et al., 2016). We assessed the impact of cisplatin oral-feeding, 1 and 9-days post blood-feeding, on *Aedes aegypti* fitness. There were no differences in mosquito mortality between the 100 μM cisplatin treated and non-treated groups (data not shown). The sole side-effect observed was a diminished egg-laying between treated groups. Cisplatin treated mosquitoes had a lowered egg production, when compared to untreated groups (Supplementary Figure 3). Visual inspection showed that cisplatin fed mosquitoes ovarian tissues were similar to the control ovarian tissue (data not shown).

**Figure 4:**
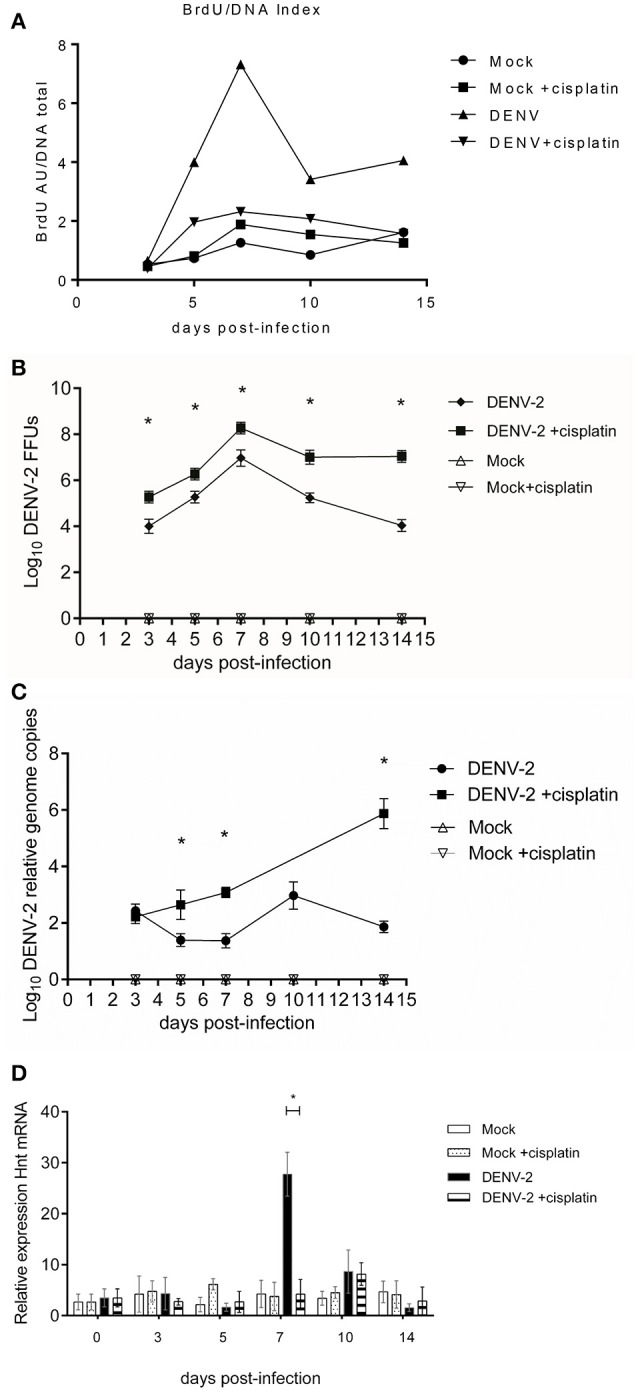
Cisplatin treated and untreated DENV-2/mock infected midguts kinetics at 3, 5, 7, 10, and 14 days post-infectious blood meal. **(A)** normalized BrdU incorporation, in Cisplatin treated and untreated DENV-2/mock infected midguts. The midguts were extracted at 3, 5, 7, 10, and 14 days post-infectious blood meal. ELISA for BrdU incorporation. Kruskal-Wallis test = 129.6, *p* < 0.0001, Mock vs. Mock +cisplatin Mann Whitney two-tailed test: non-significative difference =0.025, −0.035, 0.265, 0.735, 0.575, *p* = 0.7576, 0.0085, >0.9999, 0.7026, 0.1563, 0.3162; DENV vs. DENV +cisplatin Mann Whitney two-tailed test: all comparisons significative difference 3, 5, 7, 10, 14 dpi: −0.56, −4.70, −7.72, −4.06, −5.15; *p* = 0.0085, 0.0421, 0.0002, 0.0118, 0.0022. DENV vs. DENV +cisplatin Kolmogorov-Smirnov test: 3 and 5 dpi no-significative difference = 0.6250, 0.5, *p* = 0.0536, 0.2827. 7, 10, and 14 dpi showed significative difference = 1, 0.75, 0.75, *p* = 0.0002, 0.0186, and 0.0186. Two tests were realized for robust analysis. **(B)** Focus forming units assay of Cisplatin treated and untreated DENV-2/Mock infected midguts extracts; 3, 5, 7, 10, and 14 days post-infectious blood meal. One-way ANOVA *F* = 104.4, *p* < 0.0001. DENV-2 vs. DENV-2 cisplatin treatment, 3, 5, 7, 10, and 14 dpi Student's *T*-test with two-tailed Welch's correction: all comparison with significative difference *t* = 6.42, 5.22, 6.38, 9.8, 15.32; *p* < 0.0001. **(C)** Viral genome copies determination of Cisplatin treated and untreated DENV-2/Mock infected midguts extracts; 3, 5, 7, 10, and 14 days post-infectious blood meal. Viral genome copies. One-way ANOVA with Geisser-Greenhouse's correction epsilon = 0.1651, *F* = 170.2, *p* < 0.0001. DENV-2 vs. DENV-2+cisplatin treatment Student's *T*-test with two-tailed Welch's correction 3 dpi *t* = 1.95, *p* = 0.0679 no significative difference: 5, 7, and 14 dpi comparisons with significative difference *t* = 6.63, 16.42, 21.09; *p* < 0.0001. **(D)** Relative expression of *Hnt* transcript in the mosquito cells from Cisplatin treated and untreated DENV-2/Mock infected midguts. The kinetics were performed at 3, 5, 7, 10, and 14 days post-infectious blood meal. Mock vs. Mock +cisplatin Student's *T*-test with Welch's correction 3, 5, and 10 dpi significative difference. DENV-2 vs. DENV-2 +cisplatin Student's *T*-test with Welch's correction only 7 dpi showed significative difference *p* < 0.0001.

We determined the impact of cisplatin treatment on Dengue virus infection susceptibility of *Aedes aegypti*. Since cisplatin is known to form DNA adducts at a 100 μM concentration (thereby impeaching the DNA strands to be separated), the adduct formation would hinder DNA endoreplication. The cisplatin treated mosquitoes did show a higher viral load as well as a higher infective virus presence. Upon infection (Figures 4B,C), their DNA BrdU incorporation was diminished when compared to non-treated infected mosquitoes (Figure 4A). This experiment provides a positive link between the mosquito genomic DNA endoreplication phenomenon and the resistance to viral infection.

Also, in cisplatin treated mosquito group, *hnt* gene relative transcription was reduced to levels comparable to control groups (Figure 4D). Inhibition of *hnt* transcription would hinder the *de novo* DNA synthesis, hence inhibiting the potential antiviral mechanism hereby proposed; leaving viral replication unrestricted.

## Discussion

The genomic DNA endoreplication phenomenon has been observed in insect during development and stress response. In these conditions, endoreplication of genes related to macromolecules synthesis pathway enhance their production, hence favoring the organism growth or the cell stress resistance. This work explores the genomic response of *Aedes aegypti* to the DENV infection. Transcriptomic and microarray analysis between *Aedes aegypti* strains with high and low DENV susceptibility show a common core of active genes, indicating a functional signal transduction cascade activating the host response. Nevertheless, upon virus infection, the low-susceptibility strain showed up-regulation genes involved in several active metabolic processes (glycolysis, gluconeogenesis, cell cycle progression and differentiation). The viral resistance may be related to cell cycle alteration (Behura et al., 2011; Chauhan et al., 2012).

During viral challenge, we observed a nucleotide incorporation in the mosquitoes midguts. The inhibition of DNA replication through cisplatin treatment led to a higher virus load in the mosquitoes, thereby linking the antiviral response to the endoreplication process.

The mechanism underlying the phenomenon was explored tracking the transcription of putative *Aedes aegypti* ortholog genes of *Drosophila Delta-Notch* signaling pathway. We also quantified *hnt* transcription during the endoreplication process. We found a positive correlation between BrdU incorporation, the viral titer peak and *hnt* transcript increase.

Through unknown pathways, DENV infection actively repress the antimicrobial peptide production of *Aedes aegypti*, During the infection, the Jak/STAT, Toll, IMD and RNA interference pathways are activated in *Aedes aegypti*, supposedly through danger sensing signal cascade. This activation would provide the ability to limit viral replication and spreading in the mosquito.

There are few published works that explore global transcriptomic response between *Aedes aegypti* strains with differential vectorial competence. Hence, the mechanisms underlying these differences and ability to limit viral replication are unclear. The role of transcription factors involved in cell cycle progression, like Wnt, Hedgehog and Notch in virus inhibition has not been elucidated so far. Transcriptomics approaches only describes *Notch* transcript differences during infection; without linking viral spreading and cell cycle components (Behura et al., 2011; Chauhan et al., 2012). The Notch pathway has been implicated in host immune response against DENV in human (Li et al., 2015), so we speculated that the transcription of Notch cascade components could also be triggered in the mosquito, leading to a posterior DNA synthesis. Indeed, in *Aedes aegypti*, Delta ligand was found to be differentially over expressed during DENV infection (Colpitts et al., 2011).

Upon DENV infection, we observed increased transcription of *hnt* gene. The *hnt* gene expression is regulated by the *Delta-Notch* signaling pathway. This pathway is also transcriptionally affected by viral infection. We found that *Aedes aegypti Delta, Notch* and *hnt* transcripts were overexpressed during DENV infection. Post infection, we observed BrdU nucleotide base analog incorporation in the intestinal cells of the mosquito, implying that an active DNA replication is taking place in these cells. Since there is no cell proliferation in adult mosquitoes, we supposed that this phenomenon could be due to endoreplication process. *Hnt* protein has been described to be responsible for *Drosophila* endoreplication processes (Sun and Deng, 2007), and recent work described active transcription of the *hnt* putative gene in *Aedes albopictus* infected with DENV-2 (Tsujimoto et al., 2017). We found the *Aedes aegypti* homolog of *Aedes albopictus hindsight* and observed an over-transcription of this gene during infection. Inhibition of endoreplication process through DNA adduct formation enhanced viral infection, thereby underscoring the relevance of this cellular process in containing the viral infection.

Our results indicate that Notch pathway is activated during DENV infection in *Aedes aegypti* and endoreplication seems to be part of the mosquito response against DENV. Understanding the biological activity of Notch and its role in endoreplication of disease-transmitting mosquitoes will help to generate new strategies for controlling disease transmission. The molecular mechanisms that induce and regulate the activation of Notch pathway require further investigation.

## Author contributions

JS-S, SH-M, JM-B, RC, AA-D, FZ-E and HL-M conceived and design experiments. JS-S, SH-M, and AA-D performed the experiments. JS-S, FZ-E, and JM-B bio-informatics analysis. SH-M and HL-M contributed reagents, materials, analysis tools. JS-S, RC, and HL-M wrote the paper.

### Conflict of interest statement

The authors declare that the research was conducted in the absence of any commercial or financial relationships that could be construed as a potential conflict of interest.

## Footnotes

^1^http://www.kegg.jp/dbget-bin/www_bget?aag04330

^2^www.flybase.org

^3^www.vectorbase.org

^4^www.ensembl.org

^5^www.ncbi.nlm.nih.gov

^6^http://tree.bio.ed.ac.uk/software/figtree/
