# Supplementary material for: De Novo DNA Synthesis in Aedes aegypti Midgut Cells as a Complementary Strategy to Limit Dengue Viral Replication
Source: Front Microbiol. 2018 Apr 26;9:801. doi: 10.3389/fmicb.2018.00801 (PMC5932203; doi:10.3389/fmicb.2018.00801)
Supplement: Supplementary file 2 [file Data_Sheet_2.docx]

# Supplementary Figures

| Specie | ID genome | Putative orthologues (OrthoDB) | |
| --- | --- | --- | --- |
| *Drosophila melanogaster* | | ***Aedes aegypti*** | ***Anopheles albimanus*** |
| Delta | FBgn0000463 | AAEL011396 | AALB001278 |
| Notch Receptor | FBgn0004647 | AAEL010210 | AALB006920 |
| Hindsight | FBgn0003053 | N/A | AALB003334 *AGAP000984 |
| Cut | FBgn0004198 | AAEL007605 | AALB009392 |
| String | FBgn0003525 | AAEL014408 | AALB008080 |
| Serrate | FBgn0004197 | AAEL005082 | AALB008573 |
| Fringe | FBgn0011591 | AAEL002253 | AALB005204 |
| TACE | FBgn0039734 | AAEL003910 | AALB002747 |
| Dvl | FBgn0000499 | AAEL008234 | AALB003165 |
| Numb | FBgn0002973 | AAEL001476 | AALB007895 |
| Deltex | FBgn0000524 | AAEL001459 | N/A *AGAP000903 |
| γ-Secretase complex | |  |  |
| PSE2 | FBgn0053198 | AAEL005965 | AALB000981 |
| PSEN | FBgn0284421 | AAEL017503 | AALB008518 |
| NCSTN | FBgn0039234 | AAEL004633 | N/A *AGAP001734 |
| APH-1 | FBgn0031458 | AAEL002389 | AALB001573 |
| Co-repressor | |  |  |
| Hairless | FBgn0001169 | AAEL008617 | AALB003600 |
| CtBP | FBgn0020496 | AAEL005400 | AALB002790 |
| Groucho | FBgn0001139 | AAEL005009 | ALB002559 |
| CIR | FBgn0024483 | AAEL011667 | AALB007321 |
| HDAC | FBgn0015805 | AAEL004586 | AALB005614 |
| CSL | FBgn0004837 | AAEL006419 | AALB007981 |
| Co-activator | |  |  |
| MAML |  |  |  |
| HATs | FBgn0037376 | AAEL006177 | AALB009510 |
| SKIP | FBgn0004856 | AAEL011326 | AALB002995 |
| Anaphase Promoting Complex/cyclosome | |  |  |
| APC4 | FBgn0052707 | AAEL000116 | AALB009054 |
| APC7 | FBgn0029879 | AAEL011162 | AALB008159 |
| APC10 | FBgn0034231 | AAEL007871 | AALB010518 |
| Cdc16 | FBgn0025781 | AAEL003744 | AALB008313 |
| Cdc23 | FBgn0032863 | AAEL003273 | AALB000663 |
| Cdc27 | FBgn0012058 | AAEL014508 | AALB006241 |
| Fzy | FBgn0001086 | AAEL014025 | AALB001955 |
| Ida | FBgn0041147 | AAEL002752 | AALB004924 |
| lmgA | FBgn0250903 | AAEL007241 | AALB003214 |
| Mr | FBgn0002791 | AAEL001756 | AALB004991 |
| Rap | FBgn0262699 | AAEL004480 | AALB002606 |
| Shtd | FBgn0004391 | AAEL004064 | AALB007013 |
| Cdk1 | FBgn0004106 | AAEL008621 | AALB001637 |
| Cyc A | FBgn0000404 | AAEL000672 | AALB003801 |
| Cyc B | FBgn0000405 | AAEL010094 | AALB007534 |

**Supp. Table 1.** Main molecules of Delta-Notch signaling pathway involved in mitosis to endocycle switch in *D. melanogaster* oocyte development (Edgar et al., 2014).


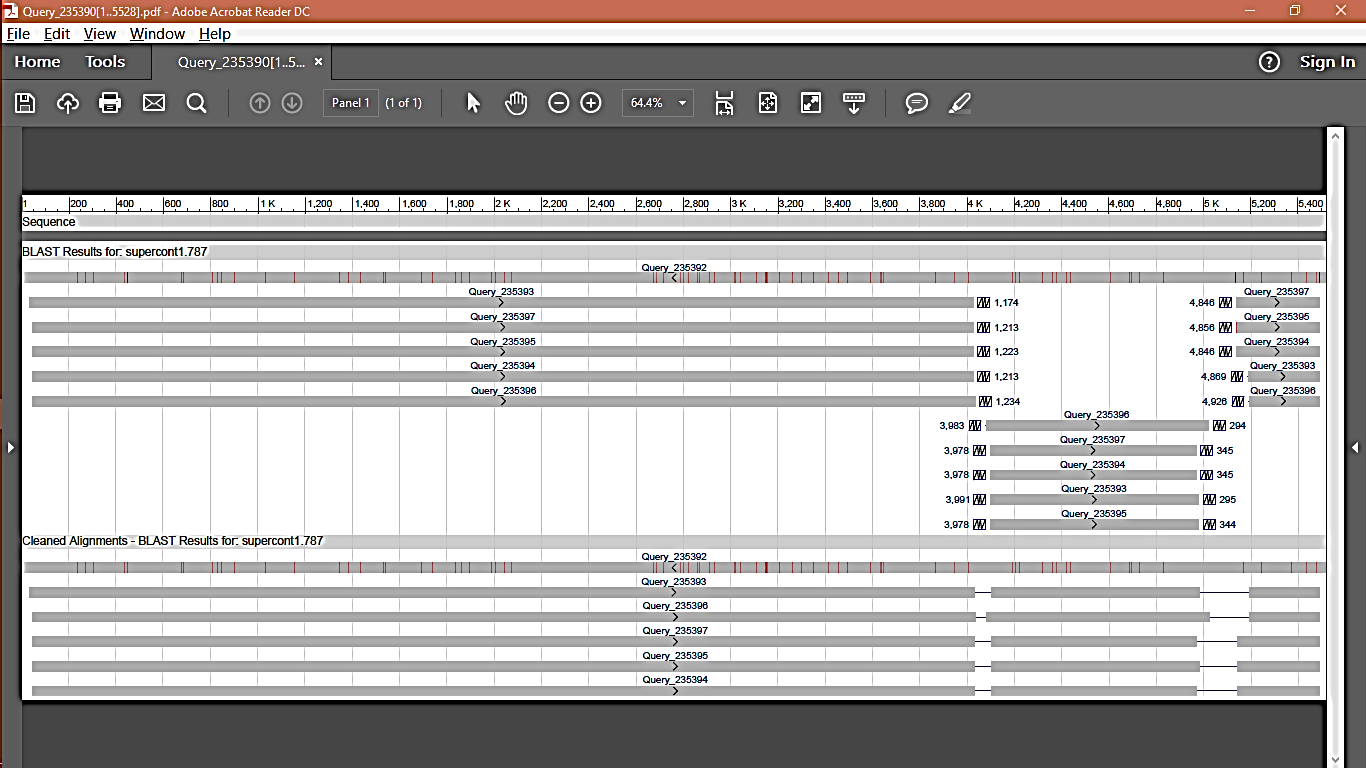

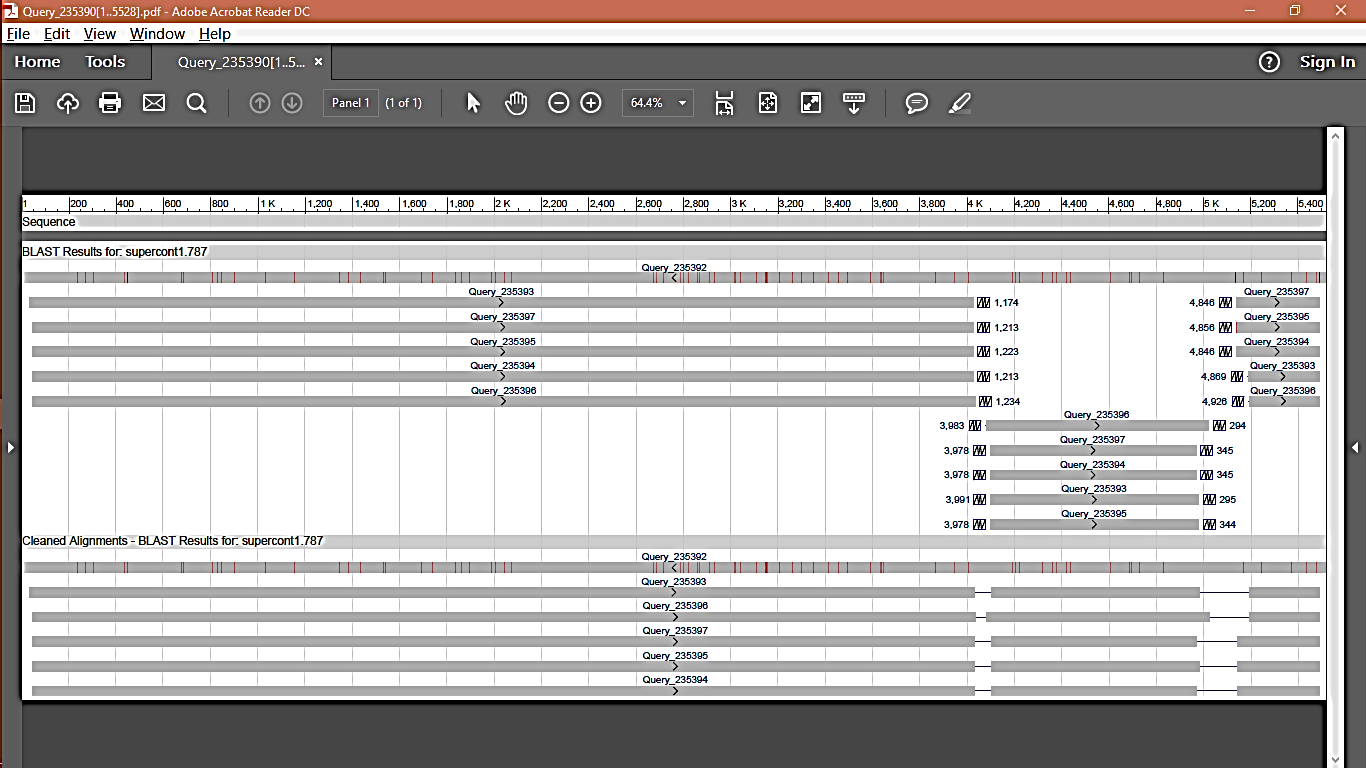


726 507 566 pbs

**Supp. Figure 1. Genomic map of the *Hindsight* gene in *Aedes aegypti* genome supercontig 1.787.** Schematic wide lines indicate the Putative protein coding region. Non-coding sequences are represented as a thin line. Two introns are predicted to be excised to form mature Hnt mRNA. The boxed areas indicate the set of oligonucleotides designed to analyse the putative gene transcription.

| Primer | Sequence (5´- 3´) | Coordinates | Amplicon size expected from DNAg | Amplicon size expected from cDNA (RNAm) |
| --- | --- | --- | --- | --- |
| Hnt A (Fwd) | ACTGCGCTCGAACATGGAG | 3186-3204 | 726 | 726 |
| Hnt A (Rev) | TCACGCTCTAACTCCTTGCG  (CGCAAGGAGTTAGAGCGTGA) | 3892-3911 |  |  |
| Hnt B (Fwd) | CGCAAGGAGTTAGAGCGTGA | 3892-3911 | 507 | 438 |
| Hnt B (Rev) | GTGTCGATCGCAGTTGGACT  (AGTCCAACTGCGATCGACAC) | 4379-4398 |  |  |
| Hnt C (Fwd) | AGTCCAACTGCGATCGACAC | 4379-4398 | 566 | 566 |
| Hnt C (Rev) | CTTTCCACCCCGACAACCTT  (AAGGTTGTCGGGGTGGAAAG) | 4925-4944 |  |  |
|  |  |  |  |  |

**Supp. Table 2. Sequences of the oligonucleotide primers used for the transcriptional analysis of the *Aedes aegypti Hindsight gene.***

| **>Aedes_aegypti_Hindsight_genomic_region_(AaegL 3.5 SUPERCONT1.787:279787:285314)**  AATCGAACTTCTTTTCCATCTTCCTCCAACAGATCACAACCCGCCATGGATCTCCGCCGG  TTGTCAGCGCACTCGGACTCCTCGCAGGAACCGATGGACATCAGCTCCGGCAATAACCGG  ACGATCAACAGCAGCAGCAGCGCCAGCGATGCCAACAACTCCTCGCTGAACAACAACAAT  CATCTCCTCAGCAAAGCCCTGAACCACCATCACCGCCATCCCAGCCAGCACCAGATGGTC  AGTGCCGACAATTCCGACAATGTCGACTCAGATGAAGACCGCCGGCAGCAATCGCGGCAC  GCCTCGGTACTTCACCAAGCCCGCCACAGCGCCCGCAGCGAGAACCTGCACGCCTTGATG  GAAACCATGCTGCGAAGGAAGGTGCACAGCTTGAACAACAACAACTACGGCAGCGGCACC  ACGCTCTTACAGCAACACCTAGCAGCAGCTGCAGCGGCCGCCAATGTCGAAAACAACAAC  CACGAACCGTCGACGGAGGGCGGCGAAGTGACCAAACGGTACGTTTGCCCCATCTGCGAC  ACAATCTCCATGACCAAACATGACTTCACGGAGCACATCCGGTCGCACAACAACAGCAAA  ACCGGCGCCTCCGGAGACAACGGAGAAGGCCAGTTTGTGTGCAAGATCTGCTCCAAGGTG  CTCTCTTCGGCGTCCTCGCTGGATCGGCACGTCCTCGTCCACACCGGAGAGCGCCCGTTC  AACTGCAAGTACTGCAATCTGACGTTCACCACCAACGGCAACATGCACCGGCACATGCGA  ACCCACAAGCAATCGGAACGGGAAAGCTACGAAAGCGACGGGTCAACGGACAGCGGAGGC  AGCAGCGGCGGAAGCAGTGGCCTTGCCAACAACAATAACAACATCTACAGCAGCAATTAC  GATGGCGAGGGAAAGCGGAAGAACTCCGATGAGAACGTGCACTACAAGCGCAAGATCCGA  ACCATCAACAACAACATCCTGGACGGAAGCGTAACCGAAGGTGTGCAGAAGTTCTGCTGC  CCGGTTTGCATTCGGAACGACTTCTCCAGCATGGTCAGCTTGGAGAATCACATGGACCGG  GAACATCCGCAGATTCCGGCCAAGTGCAGACACTGCGAGGTGGTGTTCAAAAGCTACAAG  GCCCTGAATGCCCACCGATGCGGCAACAACAACTACCAGAACATCATGCCCGGATTCAAG  GACTTGACGTTCGTGGATTTCTCGAGCGAAAAGTTCCCCCTGATCGCCAAAAGCGTCTGC  GAGCAGAGCATCCGAACTCCCGTCACCAGCCAGAAGTTCGAGTGCGACAAATGCTACCGA  GCATTCCCCTGCTCGAAGACGCTCGACATGCACATGAAGGACTGCGGCGTGTCGGACAAC  AGCGTTGTGGGAGAAAAGCGCAAATGGAAAACCAGCGAAGGTTCCTCGGAGGAAGAAGTC  AAGCGGGACGACTTCTTCGCCAATCTCGACCTGCAGAACAAATCCATGTCTACCAACATG  TCATCGAACGTATCGGAAGCCCCGACGACACCTTCTTCGTTGGACAAGTCCTTCTCCTCT  CCCATCATGTCCCGAGAGATCAAGCAGGAACCGACGACCTACTACCACCACAGCGGAGCC  AACTTCCCCGCTCAACAGGACACCAAAGATCTCGCCGACATTCAGTCGATCATCAATGTG  ACCTCCTCGGGAAGCTTCTTCCGCCAGCTGGACAAGGACCCCTACCCTCCCCTCAAGGAT  GAAGAGGAAGCCCAGGATGCCTTCACCGCCGAGTTCCGCAAGATGAAACTCCGCGGCGAG  TTCCCGTGCCGACTGTGCACCGCAGTTTTCCCGAACTTGCGCGCCCTCAAGGGACACAAC  CGCATCCACGTCTCAGCCGCCGGACCTGGACCGTACCGCTGTAACATGTGTCCGTATCTG  ATCAACGACAAAGCAACCCTCATCCGGCACATGCGAACCCACAACGGGGATCGGCCGTAC  GAGTGCGCGCTCTGCAACTACGCCTTCACAACCAAGGCCAATTGCGAGCGCCATCTCCGC  AACCGTCACGGACGGGCTAGCCGAGATGAAGTCAAGCGCGCCATCATCTACCATCCGTCA  GAGGATTCGTCTTGCGAAGATCCGCTCAAGAAGCTGCGGATGTTCGACAGCTCACCGGGA  GACTTCGATCGGGACATCGACGTGGACCATCCCTCAGATCGAAGCACTCCGGTATCTCAT  CTTAAGGACATGCTCATGCCAAGTCCGATGAACCTAGTCACCAAAATCGATAGCAACCCG  ATGCCAACTACTCCGGCCAAAATTCAGGTCAAGAGCTTGGAGAAGCTCAATCAGCTGACT  CCCCCTCAGGATCAAGACTACGAGAAGGAGATCAAACCAAAGTCCTACGGAGGTGTGCTG  GATCTCCGCAAAAAGCCGGAACCTTTGGTCGTCCGTCGGGAACCCGATGCCGACTCCGAT  CATCCACGTTCGGAACCAGAGGATGAAGAAGATGACGACGACGAGGATGATGATGGTCGC  AGGGAAGACGACGCCGAAGACGAAGACGAGGAAGAACAGCAACAGCCAGCCAAAATGCCC  AAACTGGATTTGTCTCAACTGGAAAAGAACCAACAGCAGCTGCAGTTGATGCAGCAGAAG  CTGTTCAGCGAAGCCCTCACCGATCCCACTCGTTACTTCCAGCTCAGCCAGCTGTACAGT  CGCTGCGGTTTCCCAGCGATGCCTTTCCCACTTCCACTGTTCCTCAACAATCCCCTCCTC  TGCGCACCGGGTGCCCTCGGGGACATGAAAAACTTCTTCCCCAAAGAGTTTCCCACCATG  TTACCGCAAATGTCCGGCGGAAGCCTCATCGGAAACCCATTCCTTTCGCCATCTGAGTCG  CCCAAACAGCAGGATCAGCCGCAGCCTCAGCAGTCGCCGCTGTCCGTGAACGCCAACCAC  GTTTCGCCGCTGCCGTCGTCCAAGCATGCTCAACCATCGCCGCAAGGATTGCCAAATCAC  CTTCACAAGTCTCAACCGCCGCAGGCACCACCTCCACCACCACCCCCCCAAATGCCAACG  ATTCCCCTCGGAAACGGACCCGTCAAAATGGTCATCAAGAACGGAGTCCTGATGCCAAAG  CAGAAGCAACGCCGCTACCGTACCGAACGCCCATTCGCCTGCGAACATTGTTCAGCCCGG  TTCACACTGCGCTCGAACATGGAGCGGCATATCAAGCAGCAGCATCCGCAGTACTGGTCG  CAGCGACAGCGCGGCGGCCACCACATGATGCGCGGTCGAGGAGCGAGTCATCACGCTGGG  ATGGCCGGTGCTCATCACCATCCGTCGATGCCGGCGGCCACCACTGCAGCGTCCCCCTTC  GGTAGCATCTCCGAGCAGGTCAAATACGCCATTTTGGCTCAGCAGTCGGGTAAGGCAGGA  CGCGCCGGGGAAGGAAGTGGGATGAGCAGCATGCTTCAGAACATCATTGCTCAAGGACAG  CGTCCGAACGCACCGCTGGAGCAGCACCATGACGAACATCAGCAGCGACTGCCAATGGAG  ATGCAGAAGCTGCAGCAGTTCCTGCAACAGCAGCAACAACAGGTTCTGCAACAGCATCAG  CAGCAGCAAATGGAAAGACCAAGTGCCAACCACAGCAGCACCAGCAATAACAATGGACAC  GGAGCGGATGATGAGGAAGAGGAAGATGAGCGCGAGTTGGTCATCGACGAGGAATTCCAG  CCGGAGGATTTGAGCAAGGGAAACGAATCGGAAGACGAGAGGCCGTCGAAGAAGGAGAGT  CCGGTGTTTCAGCATCAGATTCTGAAGCAGAAACTGGAGGAGAGTAAGGAGCAGAGACAG  CAAGCGGCTAAGGCTGTGGCCGAGGGGATTTTGGAGCAAGCGATGCGCCAGCGCAAGGAG  TTAGAGCGTGAGAAGGAGAAGGAAGCTGCCAAGGATGTGGACAGCGGCAAGGAGGAAGGC  GATTTGGTTCCGGTGTCCAAGCTGGTGGACAATGCAACGAACGTCGCGTTCGAGAACTAC  TTTAGGTAAATTTCGACTTTTCATGCGTAGTTAGCGTCTAAGATCTTTGGAATATTATTA  ATGAAAGGTTTTTCTTACAGCAGACCTGAGGTTCCGCTGTCGCAGGACCAGAGCGATGAG  GAGGGCTTGGTGGCGTCCGGCTCGGCTTCGGAGAGCAACAACTCCGGAACGGATGATCCC  AATCCGTCCTCGATCCAGCAGAAGAAGAAATCCGCCTACAGTTTGGCTCCGAATCGGGTC  AGCTGTCCGTACTGTCAGCGGATGTTCCCGTGGTCCAGTTCTCTACGGCGCCACATCCTC  ACGCACACCGGTCAGAAACCGTTCAAATGTTCGCAGTGCACACTGCTGTTCACTACAAAG  TCCAACTGCGATCGACACCTGCTGCGAAAGCACGGTGACGTTGAATCGGCTATGTCGATT  CCGGTGCCAATCGATGATCTGCTGGACCCGAAGCCGGAACCCATTCCGGTTGCCGTTGCC  GAAGCCATTGCCAAATCCAAAGCCACACCACCAACTTCCCGCCCAGTCAGCCCCAAGCCC  CAACCTGCTCAACCTACAGAAGAACCCAAGAAGCATGCCAAGCAACCAAAACCGGAAGAA  CCTGAACCGCTCCCAGCGCCACCGTGTCCACCGCAAATCAAAGAAGAACCCTCCGCCGAT  GAAGAGCATCCCGCGGAGCCCCACATGCCACTCGACGACCCAAGCATCCCGCCAGTCAAC  TCGGATCTCCCGTTCAAGTGCCACCTGTGCGACAGTTCCTTCGTCGATCGCGTTTCCTGC  CTGGAGCACATCAAGCTCGCTCACGCCCACGATTTTGCCCTGCTCATGAACAAGGTAACG  CTGGAGGCGGAAAGCGAAGCGCCCTCCGCCTCGCCCGACGACGACGAGAGCGGCAACAAT  GGCGAAGGTTGTCGGGGTGGAAAGTATCCCGACTACGCCAATCGGAAGGTAGGTCGCAGG  TCAGTTTTAAACAACGACGCCGACATGTGCCCTCGCGGATGAGCTGAATGTACTAATGAG  AGATTTTCTTTTTCGCCTACAGGTAATTTGCGCCTTTTGTCTGCGTCGCTTCTGGTCGAC  GGAAGATCTGAGGCGCCACATGCGTACGCATTCCCGGCGAGCGGCCCTTCAAGTGCGACG  TGTGCCAACGACGGTTCACCCTGAAGCACAGCATGCTGCGCCACCAGCGGAAGCACAAGT  GCGGTCGCATCGGCGGCATTTTGTCGAAAATTGTTGGAGCCGCCGGTGCAGCCGCCGCCG  CCGCCGCGGCCGTCCACAGCAACAGCTCCGACCTGAGCGACGACGAGCAGGACCATCCGG  CCCTGCTGTCCCGGTCCAAGCAATCGCTCCTGAGCAATTCGGATCTGATCAGCAACCTGC  TCGGCATCAACGACCAGGGCATTCTGAACCGGATGCTGCTCGGGTCCGCCTCGGAAGCGG  CCAAACTGCTCGGCGTCGAGAAGTAGGAAGAGCCGGTGCGTGCCGGCGCCGGGAAGCAGC  GATTCTAG |
| --- |
| **>Aedes_aegypti_Hindsight_putative_coding_sequence**  aatcgaacttcttttccatcttcctccaacagatcacaacccgcc  ATGGATCTCCGCCGGTTGTCAGCGCACTCGGACTCCTCGCAGGAACCGATGGACATCAGC  TCCGGCAATAACCGGACGATCAACAGCAGCAGCAGCGCCAGCGATGCCAACAACTCCTCG  CTGAACAACAACAATCATCTCCTCAGCAAAGCCCTGAACCACCATCACCGCCATCCCAGC  CAGCACCAGATGGTCAGTGCCGACAATTCCGACAATGTCGACTCAGATGAAGACCGCCGG  CAGCAATCGCGGCACGCCTCGGTACTTCACCAAGCCCGCCACAGCGCCCGCAGCGAGAAC  CTGCACGCCTTGATGGAAACCATGCTGCGAAGGAAGGTGCACAGCTTGAACAACAACAAC  TACGGCAGCGGCACCACGCTCTTACAGCAACACCTAGCAGCAGCTGCAGCGGCCGCCAAT  GTCGAAAACAACAACCACGAACCGTCGACGGAGGGCGGCGAAGTGACCAAACGGTACGTT  TGCCCCATCTGCGACACAATCTCCATGACCAAACATGACTTCACGGAGCACATCCGGTCG  CACAACAACAGCAAAACCGGCGCCTCCGGAGACAACGGAGAAGGCCAGTTTGTGTGCAAG  ATCTGCTCCAAGGTGCTCTCTTCGGCGTCCTCGCTGGATCGGCACGTCCTCGTCCACACC  GGAGAGCGCCCGTTCAACTGCAAGTACTGCAATCTGACGTTCACCACCAACGGCAACATG  CACCGGCACATGCGAACCCACAAGCAATCGGAACGGGAAAGCTACGAAAGCGACGGGTCA  ACGGACAGCGGAGGCAGCAGCGGCGGAAGCAGTGGCCTTGCCAACAACAATAACAACATC  TACAGCAGCAATTACGATGGCGAGGGAAAGCGGAAGAACTCCGATGAGAACGTGCACTAC  AAGCGCAAGATCCGAACCATCAACAACAACATCCTGGACGGAAGCGTAACCGAAGGTGTG  CAGAAGTTCTGCTGCCCGGTTTGCATTCGGAACGACTTCTCCAGCATGGTCAGCTTGGAG  AATCACATGGACCGGGAACATCCGCAGATTCCGGCCAAGTGCAGACACTGCGAGGTGGTG  TTCAAAAGCTACAAGGCCCTGAATGCCCACCGATGCGGCAACAACAACTACCAGAACATC  ATGCCCGGATTCAAGGACTTGACGTTCGTGGATTTCTCGAGCGAAAAGTTCCCCCTGATC  GCCAAAAGCGTCTGCGAGCAGAGCATCCGAACTCCCGTCACCAGCCAGAAGTTCGAGTGC  GACAAATGCTACCGAGCATTCCCCTGCTCGAAGACGCTCGACATGCACATGAAGGACTGC  GGCGTGTCGGACAACAGCGTTGTGGGAGAAAAGCGCAAATGGAAAACCAGCGAAGGTTCC  TCGGAGGAAGAAGTCAAGCGGGACGACTTCTTCGCCAATCTCGACCTGCAGAACAAATCC  ATGTCTACCAACATGTCATCGAACGTATCGGAAGCCCCGACGACACCTTCTTCGTTGGAC  AAGTCCTTCTCCTCTCCCATCATGTCCCGAGAGATCAAGCAGGAACCGACGACCTACTAC  CACCACAGCGGAGCCAACTTCCCCGCTCAACAGGACACCAAAGATCTCGCCGACATTCAG  TCGATCATCAATGTGACCTCCTCGGGAAGCTTCTTCCGCCAGCTGGACAAGGACCCCTAC  CCTCCCCTCAAGGATGAAGAGGAAGCCCAGGATGCCTTCACCGCCGAGTTCCGCAAGATG  AAACTCCGCGGCGAGTTCCCGTGCCGACTGTGCACCGCAGTTTTCCCGAACTTGCGCGCC  CTCAAGGGACACAACCGCATCCACGTCTCAGCCGCCGGACCTGGACCGTACCGCTGTAAC  ATGTGTCCGTATCTGATCAACGACAAAGCAACCCTCATCCGGCACATGCGAACCCACAAC  GGGGATCGGCCGTACGAGTGCGCGCTCTGCAACTACGCCTTCACAACCAAGGCCAATTGC  GAGCGCCATCTCCGCAACCGTCACGGACGGGCTAGCCGAGATGAAGTCAAGCGCGCCATC  ATCTACCATCCGTCAGAGGATTCGTCTTGCGAAGATCCGCTCAAGAAGCTGCGGATGTTC  GACAGCTCACCGGGAGACTTCGATCGGGACATCGACGTGGACCATCCCTCAGATCGAAGC  ACTCCGGTATCTCATCTTAAGGACATGCTCATGCCAAGTCCGATGAACCTAGTCACCAAA  ATCGATAGCAACCCGATGCCAACTACTCCGGCCAAAATTCAGGTCAAGAGCTTGGAGAAG  CTCAATCAGCTGACTCCCCCTCAGGATCAAGACTACGAGAAGGAGATCAAACCAAAGTCC  TACGGAGGTGTGCTGGATCTCCGCAAAAAGCCGGAACCTTTGGTCGTCCGTCGGGAACCC  GATGCCGACTCCGATCATCCACGTTCGGAACCAGAGGATGAAGAAGATGACGACGACGAG  GATGATGATGGTCGCAGGGAAGACGACGCCGAAGACGAAGACGAGGAAGAACAGCAACAG  CCAGCCAAAATGCCCAAACTGGATTTGTCTCAACTGGAAAAGAACCAACAGCAGCTGCAG  TTGATGCAGCAGAAGCTGTTCAGCGAAGCCCTCACCGATCCCACTCGTTACTTCCAGCTC  AGCCAGCTGTACAGTCGCTGCGGTTTCCCAGCGATGCCTTTCCCACTTCCACTGTTCCTC  AACAATCCCCTCCTCTGCGCACCGGGTGCCCTCGGGGACATGAAAAACTTCTTCCCCAAA  GAGTTTCCCACCATGTTACCGCAAATGTCCGGCGGAAGCCTCATCGGAAACCCATTCCTT  TCGCCATCTGAGTCGCCCAAACAGCAGGATCAGCCGCAGCCTCAGCAGTCGCCGCTGTCC  GTGAACGCCAACCACGTTTCGCCGCTGCCGTCGTCCAAGCATGCTCAACCATCGCCGCAA  GGATTGCCAAATCACCTTCACAAGTCTCAACCGCCGCAGGCACCACCTCCACCACCACCC  CCCCAAATGCCAACGATTCCCCTCGGAAACGGACCCGTCAAAATGGTCATCAAGAACGGA  GTCCTGATGCCAAAGCAGAAGCAACGCCGCTACCGTACCGAACGCCCATTCGCCTGCGAA  CATTGTTCAGCCCGGTTCACACTGCGCTCGAACATGGAGCGGCATATCAAGCAGCAGCAT  CCGCAGTACTGGTCGCAGCGACAGCGCGGCGGCCACCACATGATGCGCGGTCGAGGAGCG  AGTCATCACGCTGGGATGGCCGGTGCTCATCACCATCCGTCGATGCCGGCGGCCACCACT  GCAGCGTCCCCCTTCGGTAGCATCTCCGAGCAGGTCAAATACGCCATTTTGGCTCAGCAG  TCGGGTAAGGCAGGACGCGCCGGGGAAGGAAGTGGGATGAGCAGCATGCTTCAGAACATC  ATTGCTCAAGGACAGCGTCCGAACGCACCGCTGGAGCAGCACCATGACGAACATCAGCAG  CGACTGCCAATGGAGATGCAGAAGCTGCAGCAGTTCCTGCAACAGCAGCAACAACAGGTT  CTGCAACAGCATCAGCAGCAGCAAATGGAAAGACCAAGTGCCAACCACAGCAGCACCAGC  AATAACAATGGACACGGAGCGGATGATGAGGAAGAGGAAGATGAGCGCGAGTTGGTCATC  GACGAGGAATTCCAGCCGGAGGATTTGAGCAAGGGAAACGAATCGGAAGACGAGAGGCCG  TCGAAGAAGGAGAGTCCGGTGTTTCAGCATCAGATTCTGAAGCAGAAACTGGAGGAGAGT  AAGGAGCAGAGACAGCAAGCGGCTAAGGCTGTGGCCGAGGGGATTTTGGAGCAAGCGATG  CGCCAGCGCAAGGAGTTAGAGCGTGAGAAGGAGAAGGAAGCTGCCAAGGATGTGGACAGC  GGCAAGGAGGAAGGCGATTTGGTTCCGGTGTCCAAGCTGGTGGACAATGCAACGAACGTC  GCGTTCGAGAACTACTTT  aggtaaatttcgacttttcatgcgtagttagcgtctaagatctttggaatattattaatg  aaaggtttttcttac  AGCAGACCTGAGGTTCCGCTGTCGCAGGACCAGAGCGATGAGGAGGGCTTGGTGGCGTCC  GGCTCGGCTTCGGAGAGCAACAACTCCGGAACGGATGATCCCAATCCGTCCTCGATCCAG  CAGAAGAAGAAATCCGCCTACAGTTTGGCTCCGAATCGGGTCAGCTGTCCGTACTGTCAG  CGGATGTTCCCGTGGTCCAGTTCTCTACGGCGCCACATCCTCACGCACACCGGTCAGAAA  CCGTTCAAATGTTCGCAGTGCACACTGCTGTTCACTACAAAGTCCAACTGCGATCGACAC  CTGCTGCGAAAGCACGGTGACGTTGAATCGGCTATGTCGATTCCGGTGCCAATCGATGAT  CTGCTGGACCCGAAGCCGGAACCCATTCCGGTTGCCGTTGCCGAAGCCATTGCCAAATCC  AAAGCCACACCACCAACTTCCCGCCCAGTCAGCCCCAAGCCCCAACCTGCTCAACCTACA  GAAGAACCCAAGAAGCATGCCAAGCAACCAAAACCGGAAGAACCTGAACCGCTCCCAGCG  CCACCGTGTCCACCGCAAATCAAAGAAGAACCCTCCGCCGATGAAGAGCATCCCGCGGAG  CCCCACATGCCACTCGACGACCCAAGCATCCCGCCAGTCAACTCGGATCTCCCGTTCAAG  TGCCACCTGTGCGACAGTTCCTTCGTCGATCGCGTTTCCTGCCTGGAGCACATCAAGCTC  GCTCACGCCCACGATTTTGCCCTGCTCATGAACAAGGTAACGCTGGAGGCGGAAAGCGAA  GCGCCCTCCGCCTCGCCCGACGACGACGAGAGCGGCAACAATGGCGAAGGTTGTCGGGGT  GGAAAGTATCCCGACTACGCCAATCGGA  aggtaggtcgcaggtcagttttaaacaacgacgccgacatgtgccctcgcggatgagctg  aatgtactaatgagagattttctttttcgcctacaggtaatttgcgccttttgtctgcgt  cgcttctggtcgacggaagatctgaggcgccacatgcgtacgcattcccggcg  AGCGGCCCTTCAAGTGCGACGTGTGCCAACGACGGTTCACCCTGAAGCACAGCATGCTGC  GCCACCAGCGGAAGCACAAGTGCGGTCGCATCGGCGGCATTTTGTCGAAAATTGTTGGAG  CCGCCGGTGCAGCCGCCGCCGCCGCCGCGGCCGTCCACAGCAACAGCTCCGACCTGAGCG  ACGACGAGCAGGACCATCCGGCCCTGCTGTCCCGGTCCAAGCAATCGCTCCTGAGCAATT  CGGATCTGATCAGCAACCTGCTCGGCATCAACGACCAGGGCATTCTGAACCGGATGCTGC  TCGGGTCCGCCTCGGAAGCGGCCAAACTGCTCGGCGTCGAGAAGTAG  gaagagccggtgcgtgccggcgccgggaagcagcgattctag |
| **>Aedes_aegypti_Hindsight_putative_protein**  MDLRRLSAHSDSSQEPMDISSGNNRTINSSSSASDANNSSLNNNNHLLSKALNHHHRHPS  QHQMVSADNSDNVDSDEDRRQQSRHASVLHQARHSARSENLHALMETMLRRKVHSLNNNN  YGSGTTLLQQHLAAAAAAANVENNNHEPSTEGGEVTKRYVCPICDTISMTKHDFTEHIRS  HNNSKTGASGDNGEGQFVCKICSKVLSSASSLDRHVLVHTGERPFNCKYCNLTFTTNGNM  HRHMRTHKQSERESYESDGSTDSGGSSGGSSGLANNNNNIYSSNYDGEGKRKNSDENVHY  KRKIRTINNNILDGSVTEGVQKFCCPVCIRNDFSSMVSLENHMDREHPQIPAKCRHCEVV  FKSYKALNAHRCGNNNYQNIMPGFKDLTFVDFSSEKFPLIAKSVCEQSIRTPVTSQKFEC  DKCYRAFPCSKTLDMHMKDCGVSDNSVVGEKRKWKTSEGSSEEEVKRDDFFANLDLQNKS  MSTNMSSNVSEAPTTPSSLDKSFSSPIMSREIKQEPTTYYHHSGANFPAQQDTKDLADIQ  SIINVTSSGSFFRQLDKDPYPPLKDEEEAQDAFTAEFRKMKLRGEFPCRLCTAVFPNLRA  LKGHNRIHVSAAGPGPYRCNMCPYLINDKATLIRHMRTHNGDRPYECALCNYAFTTKANC  ERHLRNRHGRASRDEVKRAIIYHPSEDSSCEDPLKKLRMFDSSPGDFDRDIDVDHPSDRS  TPVSHLKDMLMPSPMNLVTKIDSNPMPTTPAKIQVKSLEKLNQLTPPQDQDYEKEIKPKS  YGGVLDLRKKPEPLVVRREPDADSDHPRSEPEDEEDDDDEDDDGRREDDAEDEDEEEQQQ  PAKMPKLDLSQLEKNQQQLQLMQQKLFSEALTDPTRYFQLSQLYSRCGFPAMPFPLPLFL  NNPLLCAPGALGDMKNFFPKEFPTMLPQMSGGSLIGNPFLSPSESPKQQDQPQPQQSPLS  VNANHVSPLPSSKHAQPSPQGLPNHLHKSQPPQAPPPPPPPQMPTIPLGNGPVKMVIKNG  VLMPKQKQRRYRTERPFACEHCSARFTLRSNMERHIKQQHPQYWSQRQRGGHHMMRGRGA  SHHAGMAGAHHHPSMPAATTAASPFGSISEQVKYAILAQQSGKAGRAGEGSGMSSMLQNI  IAQGQRPNAPLEQHHDEHQQRLPMEMQKLQQFLQQQQQQVLQQHQQQQMERPSANHSSTS  NNNGHGADDEEEEDERELVIDEEFQPEDLSKGNESEDERPSKKESPVFQHQILKQKLEES  KEQRQQAAKAVAEGILEQAMRQRKELEREKEKEAAKDVDSGKEEGDLVPVSKLVDNATNV  AFENYFSRPEVPLSQDQSDEEGLVASGSASESNNSGTDDPNPSSIQQKKKSAYSLAPNRV  SCPYCQRMFPWSSSLRRHILTHTGQKPFKCSQCTLLFTTKSNCDRHLLRKHGDVESAMSI  PVPIDDLLDPKPEPIPVAVAEAIAKSKATPPTSRPVSPKPQPAQPTEEPKKHAKQPKPEE  PEPLPAPPCPPQIKEEPSADEEHPAEPHMPLDDPSIPPVNSDLPFKCHLCDSSFVDRVSC  LEHIKLAHAHDFALLMNKVTLEAESEAPSASPDDDESGNNGEGCRGGKYPDYANRKRPFK  CDVCQRRFTLKHSMLRHQRKHKCGRIGGILSKIVGAAGAAAAAAAAVHSNSSDLSDDEQD  HPALLSRSKQSLLSNSDLISNLLGINDQGILNRMLLGSASEAAKLLGVEK |

**Supp. Table 3. Putative *Hnt* Genomic Sequence in *Aedes aegypti*. AaegL 3.5 SUPERCONT1.787:279787:285314, mRNA CDS and protein sequence.**

**Supp.**
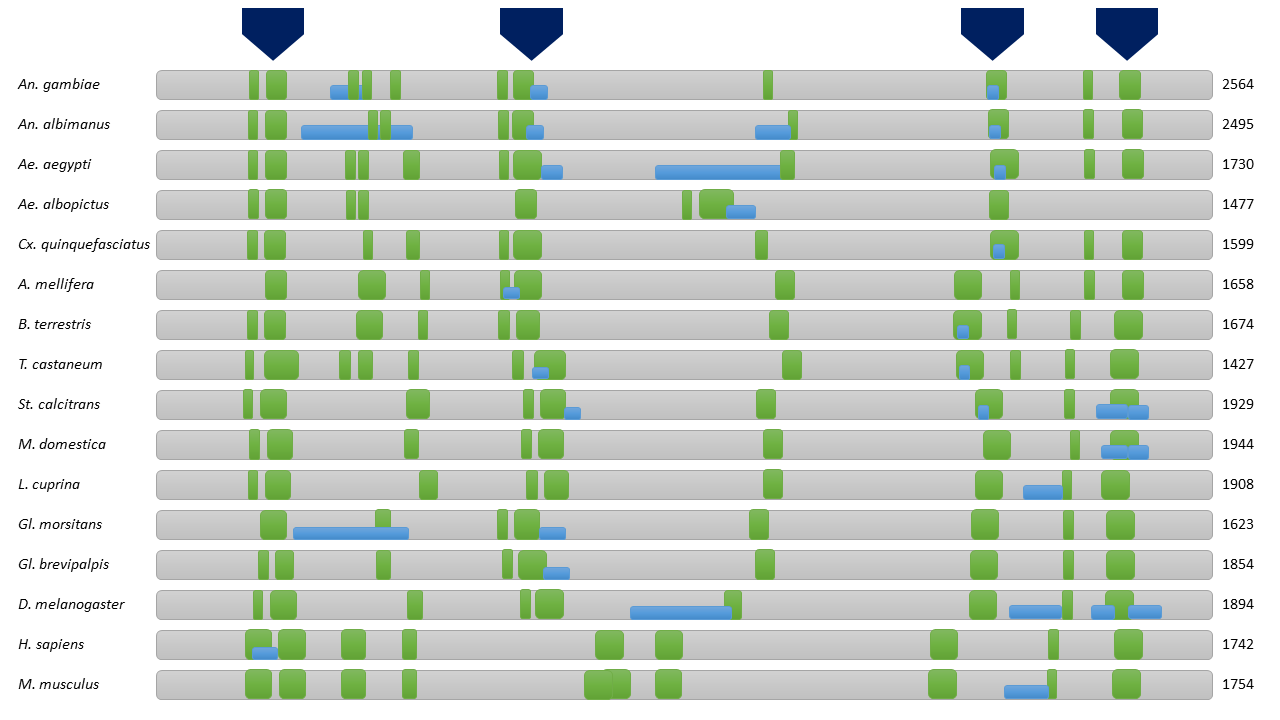
**Figure 2. Protein architecture, domains and features of the *hindsight* putative protein:** Aminoacidic sequences were analyzed with Interpro, domain and features were grouped using GBlocks tool. Green blocks represent zinc finger domains, wider green blocks mean double domain. Light blue rectangles indicate DNA binding domains which are diversely positioned. Left numbers indicate total aminoacid number in the specie. Top four dark blue arrows pointing clusters of double zinc finger domain. *Hnt* and *RREB-1* proteins families are involved in multiple development roles during ontogeny. The conserved domains of this protein family were aligned using GBlocks tool. Four clusters of double zinc finger domain are conserved in the *hnt* and *RREB* protein family, this may be considered as a signature of the protein.


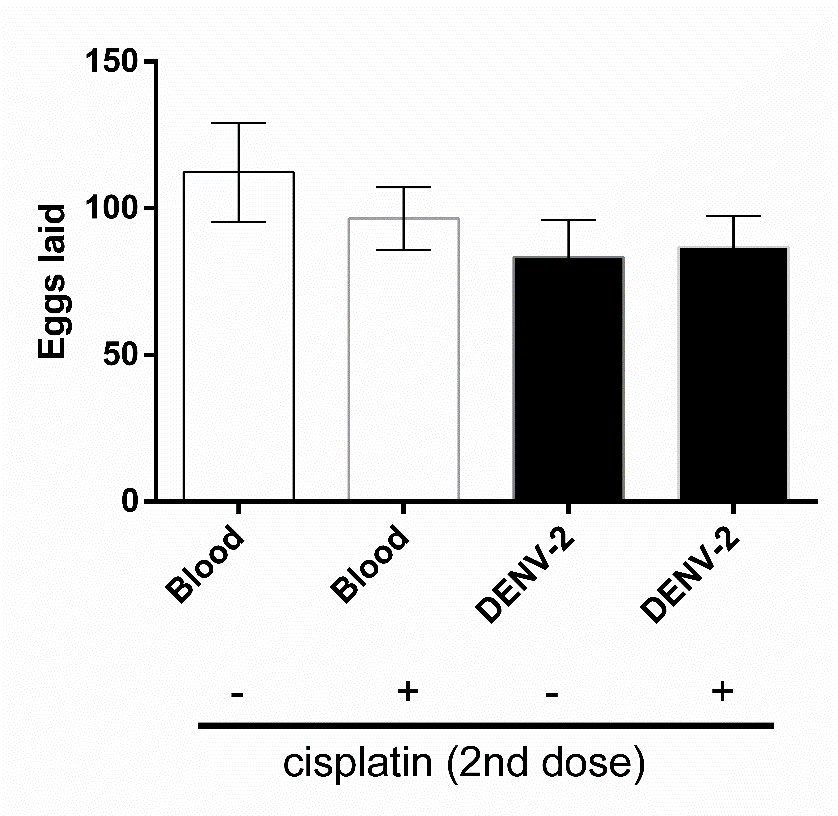

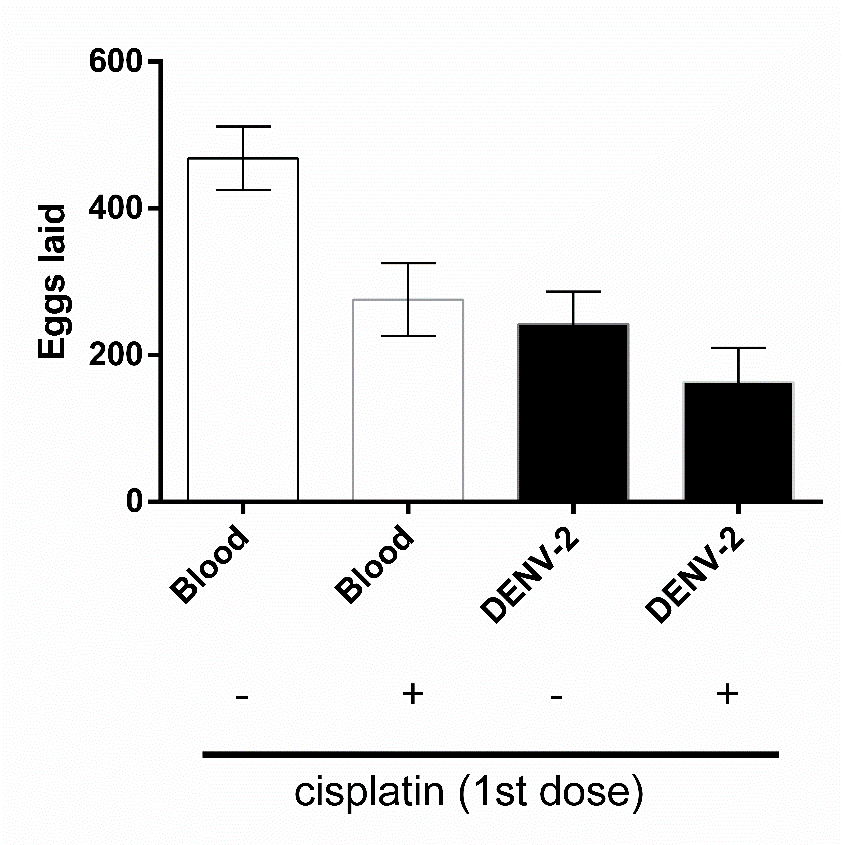


**Supp. Figure 3. Number of eggs laid post-blood feeding (with and without DENV-2) and cisplatin effect.**
